# TRIPOD+AI statement: updated guidance for reporting clinical prediction models that use regression or machine learning methods

**DOI:** 10.1136/bmj-2023-078378

**Published:** 2024-04-16

**Authors:** Gary S Collins, Karel G M Moons, Paula Dhiman, Richard D Riley, Andrew L Beam, Ben Van Calster, Marzyeh Ghassemi, Xiaoxuan Liu, Johannes B Reitsma, Maarten van Smeden, Anne-Laure Boulesteix, Jennifer Catherine Camaradou, Leo Anthony Celi, Spiros Denaxas, Alastair K Denniston, Ben Glocker, Robert M Golub, Hugh Harvey, Georg Heinze, Michael M Hoffman, André Pascal Kengne, Emily Lam, Naomi Lee, Elizabeth W Loder, Lena Maier-Hein, Bilal A Mateen, Melissa D McCradden, Lauren Oakden-Rayner, Johan Ordish, Richard Parnell, Sherri Rose, Karandeep Singh, Laure Wynants, Patricia Logullo

**Affiliations:** 1 Centre for Statistics in Medicine, UK EQUATOR Centre, Nuffield Department of Orthopaedics, Rheumatology, and Musculoskeletal Sciences, University of Oxford, Oxford OX3 7LD, UK; 2 Julius Centre for Health Sciences and Primary Care, University Medical Centre Utrecht, Utrecht University, Utrecht, Netherlands; 3 Institute of Applied Health Research, College of Medical and Dental Sciences, University of Birmingham, Birmingham, UK; 4 National Institute for Health and Care Research (NIHR) Birmingham Biomedical Research Centre, Birmingham, UK; 5 Department of Epidemiology, Harvard T H Chan School of Public Health, Boston, MA, USA; 6 Department of Development and Regeneration, KU Leuven, Leuven, Belgium; 7 Department of Biomedical Data Science, Leiden University Medical Centre, Leiden, Netherlands; 8 Department of Electrical Engineering and Computer Science, Institute for Medical Engineering and Science, Massachusetts Institute of Technology, Cambridge, MA, USA; 9 Institute of Inflammation and Ageing, College of Medical and Dental Sciences, University of Birmingham, Birmingham, UK; 10 University Hospitals Birmingham NHS Foundation Trust, Birmingham, UK; 11 Institute for Medical Information Processing, Biometry and Epidemiology, Faculty of Medicine, Ludwig-Maximilians-University of Munich and Munich Centre of Machine Learning, Germany; 12 Patient representative, Health Data Research UK patient and public involvement and engagement group; 13 Patient representative, University of East Anglia, Faculty of Health Sciences, Norwich Research Park, Norwich, UK; 14 Beth Israel Deaconess Medical Center, Boston, MA, USA; 15 Laboratory for Computational Physiology, Massachusetts Institute of Technology, Cambridge, MA, USA; 16 Department of Biostatistics, Harvard T H Chan School of Public Health, Boston, MA, USA; 17 Institute of Health Informatics, University College London, London, UK; 18 British Heart Foundation Data Science Centre, London, UK; 19 Department of Computing, Imperial College London, London, UK; 20 Northwestern University Feinberg School of Medicine, Chicago, IL, USA; 21 Hardian Health, Haywards Heath, UK; 22 Section for Clinical Biometrics, Centre for Medical Data Science, Medical University of Vienna, Vienna, Austria; 23 Princess Margaret Cancer Centre, University Health Network, Toronto, ON, Canada; 24 Department of Medical Biophysics, University of Toronto, Toronto, ON, Canada; 25 Department of Computer Science, University of Toronto, Toronto, ON, Canada; 26 Vector Institute for Artificial Intelligence, Toronto, ON, Canada; 27 Department of Medicine, University of Cape Town, Cape Town, South Africa; 28 National Institute for Health and Care Excellence, London, UK; 29 *The BMJ*, London, UK; 30 Department of Neurology, Brigham and Women’s Hospital, Harvard Medical School, Boston, MA, USA; 31 Department of Intelligent Medical Systems, German Cancer Research Centre, Heidelberg, Germany; 32 Wellcome Trust, London, UK; 33 Alan Turing Institute, London, UK; 34 Department of Bioethics, Hospital for Sick Children Toronto, ON, Canada; 35 Genetics and Genome Biology, SickKids Research Institute, Toronto, ON, Canada; 36 Australian Institute for Machine Learning, University of Adelaide, Adelaide, SA, Australia; 37 Medicines and Healthcare products Regulatory Agency, London, UK; 38 Department of Health Policy and Center for Health Policy, Stanford University, Stanford, CA, USA; 39 Department of Learning Health Sciences, University of Michigan Medical School, Ann Arbor, MI, USA; 40 Department of Epidemiology, CAPHRI Care and Public Health Research Institute, Maastricht University, Maastricht, Netherlands

## Abstract

The TRIPOD (Transparent Reporting of a multivariable prediction model for Individual Prognosis Or Diagnosis) statement was published in 2015 to provide the minimum reporting recommendations for studies developing or evaluating the performance of a prediction model. Methodological advances in the field of prediction have since included the widespread use of artificial intelligence (AI) powered by machine learning methods to develop prediction models. An update to the TRIPOD statement is thus needed. TRIPOD+AI provides harmonised guidance for reporting prediction model studies, irrespective of whether regression modelling or machine learning methods have been used. The new checklist supersedes the TRIPOD 2015 checklist, which should no longer be used. This article describes the development of TRIPOD+AI and presents the expanded 27 item checklist with more detailed explanation of each reporting recommendation, and the TRIPOD+AI for Abstracts checklist. TRIPOD+AI aims to promote the complete, accurate, and transparent reporting of studies that develop a prediction model or evaluate its performance. Complete reporting will facilitate study appraisal, model evaluation, and model implementation.

Prediction models are used across different healthcare settings. They are used to estimate an outcome value or risk. Most models estimate the probability of the presence of a particular health condition (diagnostic) or whether a particular outcome will occur in the future (prognostic).^1^ Their primary use is to support clinical decision making, such as whether to refer patients for further testing, monitor disease deterioration or treatment effects, or initiate treatment or lifestyle changes. Examples of well known prediction models include EuroSCORE II (cardiac surgery),^2^ the Gail model (breast cancer),^3^ the Framingham risk score (cardiovascular disease),^4^ IMPACT (traumatic brain injury),^5^ and FRAX (osteoporotic and hip fractures).^6^

Prediction models are abundant in the biomedical literature, with thousands of models published annually (and increasing), and have been developed for many outcomes and health conditions.^7^ ^8^ At least 731 diagnostic and prognostic prediction model studies on covid-19 were published during the first 12 months of the pandemic.^9^ Despite this interest in developing prediction models, there have been longstanding concerns about transparency and completeness of reporting in the field,^10^ ^11^ and the resulting usability. For readers (including peer reviewers, editors, health professionals, regulators, patients, and the general public), incomplete or inaccurate reporting impairs the ability to critically appraise the study design and methods, have confidence in the findings, and further evaluate or implement a prediction model. Poor reporting of a model might also mask flaws in the design, data collection, or conduct of a study that, if the model was implemented in the clinical pathway, could cause harm. Harm can be perceived to occur when insufficient measures are in place to mitigate bias. Better reporting can create more trust and influence patient and public acceptability of the use of prediction models in healthcare. Authors have an ethical and scientific obligation to honestly report their research in a complete and transparent manner. As noted by the late Doug Altman and colleagues, “Good reporting is not an optional extra; it is an essential component of research”^12^—anything less is little more than avoidable research waste.^13^

In response to concerns about incomplete reporting,^10^ ^11^ ^14^ ^15^ the TRIPOD (Transparent Reporting of a multivariable model for Individual Prognosis Or Diagnosis) statement was published in 2015 (TRIPOD 2015) to provide minimum reporting recommendations.^16^ ^17^ TRIPOD 2015 comprises a checklist of 37 items, which includes 25 items to report in both development and validation studies, and an additional six items for model development studies and six items for validation studies. Accompanying the checklist is an explanation and elaboration document that provides the rationale behind each reporting item; published examples of good reporting; and a discussion of issues relating to the design, conduct, and analysis of prediction model studies.^17^ TRIPOD 2015 mainly focused on models developed using regression modelling, which was the prevailing approach at the time. Additional guidance has since been created for reporting abstracts of prediction model studies (TRIPOD for Abstracts^18^), studies developing or validating prediction models using clustered data (TRIPOD-Cluster^19^ ^20^), systematic reviews and meta-analyses of prediction model studies (TRIPOD-SRMA^21^), and guidance in preparation for study protocols (TRIPOD-P^22^). All available guidance, as well as template checklists for filling out separately, can also be found on the TRIPOD website (https://www.tripod-statement.org/).

Since the publication of TRIPOD 2015, there have been numerous methodological advances in prediction modelling, including sample size guidance for developing models^23^ ^24^ ^25^ ^26^ ^27^ and evaluating their performance,^28^ ^29^ ^30^ ^31^ ^32^ and greater recognition of operationalising fairness,^33^ reproducibility,^34^ and adopting open science principles.^35^ However, interest and financial investment in applying methods ascribed to artificial intelligence (AI), typically powered by advances in machine learning methods (eg, random forests, deep learning), is where we have seen the most progress and change. With increasing access to data and availability of off-the-shelf software to apply machine learning methods, developing a prediction model has become faster and easier. Vast numbers of prediction models are now entering the scientific literature for many clinical settings, and for a wide range of outcomes and health conditions, with multiple models often available for the same outcome, health condition, and target population.^7^ ^8^ ^36^ The ability to critically evaluate the quality of prediction models and understand their ability to serve well in a particular setting or for a particular use case is therefore of even greater critical importance. This ability is predicated on complete and transparent reporting.

However, systematic reviews evaluating studies of prediction models have shown that they are often poorly conducted (including deficiencies in study design or data collection^37^ ^38^); use poor methodology^37^ ^38^; are incompletely reported with key details missing^39^ ^40^ ^41^ ^42^ ^43^ ^44^ ^45^ ^46^ ^47^ ^48^ ^49^ ^50^ ^51^ ^52^ ^53^ ^54^; are consequently at high risk of bias^41^ ^49^ ^55^ ^56^ ^57^; rarely adhere to open science practices,^58^ and are susceptible to overinterpretation or so-called spin.^59^ ^60^ These deficiencies cast considerable doubt on models’ usefulness and safety, and raises concerns about their potential to create or widen healthcare disparities.^61^ While TRIPOD 2015 is largely agnostic to the type of modelling approach, and much of its reporting recommendations apply equally to non-regression approaches, additional reporting considerations are needed for the growing class of machine learning methods. For example, unlike regression based models, the flexibility and complexity underpinning other machine learning approaches typically means that the resulting prediction models do not result in a simple equation and sometimes even the predictors used remain unclear. Additional reporting considerations are therefore needed that are not currently covered in TRIPOD 2015. Alongside methodological advancements, considerations of fairness,^62^ wider acceptance of open science practices,^63^ and public and patient involvement in research and implementation of research,^64^ ^65^ an update to the TRIPOD 2015 statement is needed to capture these developments and the consequences for reporting.

The aim of this paper is to describe the development of the updated TRIPOD guidance, present the new TRIPOD+AI checklist, and discuss how to use it. TRIPOD+AI aims to harmonise the landscape of prediction model studies and provide guidance regardless of whether regression models or machine learning methods have been used.^66^ The “+” in TRIPOD+AI indicates that it provides consolidated reporting recommendations for studies of prediction models developed using regression modelling or machine learning (ie, deep learning, random forests) approaches. We also use the additional term “AI” to be consistent with existing reporting guidelines for studies broadly labelled as involving AI. However, for readability, this article will refer to the methods underpinning them as machine learning (table 1). A glossary of terms (box 1) clarifies key concepts used within the TRIPOD+AI reporting guideline. 

**Table 1:** Reporting guidelines for healthcare studies using machine learning

Reporting guideline	Scope
STARD-AI	Studies evaluating the diagnostic accuracy of an artificial intelligence based test (in preparation)^67^
TRIPOD+AI	Studies developing or evaluating the performance of a prediction model, using artificial intelligence, including machine learning methods
CLAIM	Medical imaging studies using artificial intelligence^68^
DECIDE-AI	Early stage clinical evaluation (including safety, human factors evaluation) of decision support systems driven by artificial intelligence^69^
CHEERS-AI	Studies describing health economic evaluations to estimate the value for money (cost effectiveness) of artificial intelligence interventions^70^
SPIRIT-AI	Protocols for clinical trials evaluating an intervention with an artificial intelligence component^71^
CONSORT-AI	Clinical trial reports evaluating an intervention with an artificial intelligence component^72^
PRISMA-AI	Systematic reviews and meta-analyses of artificial intelligence interventions (in preparation)^73^

STARD=Standards for Reporting of Diagnostic Accuracy; TRIPOD=Transparent Reporting of a multivariable prediction model for Individual Prognosis Or Diagnosis; AI=artificial intelligence; CLAIM=Checklist for Artificial Intelligence in Medical Imaging; DECIDE=Decisions in health Care to Introduce or Diffuse innovations using Evidence; CHEERS=Consolidated Health Economic Evaluation Reporting Standards; SPIRIT=Standard Protocol Items: Recommendations for Interventional Trials; CONSORT=Consolidated Standards of Reporting Trials; PRISMA=Preferred Reporting Items for Systematic Reviews and Meta-Analyses.

Box 1 **Glossary of terms used in TRIPOD+AI**

The definitions and descriptions given below relate to the specific context of the TRIPOD+AI* guideline; they do not necessarily apply to other areas of research.

**Artificial intelligence**

Field of computer science that focuses on developing models and algorithms capable of performing tasks that typically require human intelligence.

**Calibration**

Agreement between observed outcomes and estimated values from the model. Calibration is best assessed graphically with a plot of the estimated values on the x axis and observed values on the y axis, with a smoothed flexible calibration curve in the individual data.

**Class imbalance**

When the frequency of individuals with and without the outcome event is unequal.

**Care pathway**

Structured and coordinated plan of care for managing a specific health condition or dealing with a patient’s healthcare needs throughout their healthcare journey.

**Discrimination**

How well the predictions from the model differentiate between individuals with and without the outcome. Discrimination is typically quantified by the c statistic (sometimes referred to as the area under the curve (AUC) or area under the receiver operating characteristics (AUROC)) for binary outcomes, and the c index for time-to-event outcomes.

**Evaluation or test data**

Data used to estimate the performance of a prediction model, sometimes referred to as test data or validation data.† Evaluation data should be distinct from the data used to train the model, tune hyperparameters, or do model selection, such that there is no overlap in participants between the training and evaluation data. Evaluation data should be representative of the population in whom the model is to be used.

**Fairness**

Property of prediction models that do not discriminate against individuals or groups of individuals based on attributes such as age, race/ethnicity, sex/gender, or socioeconomic status.

**Hyperparameters**

Values that control the model development or learning process.

**Hyperparameter tuning**

Finding the best (hyper)parameter settings for a particular model building strategy.

**Internal validation**

Evaluating the performance of a prediction model on the same population on which the model was developed (eg, train test split, cross validation, or bootstrapping).

**Machine learning**

A subfield of artificial intelligence that focuses on developing models capable of learning and making predictions or decisions from data, without being explicitly programmed.

**Model evaluation**

Evaluating predictive accuracy of a model by estimating model discrimination (eg, c statistic), model calibration (eg, calibration plot, calibration slope), and clinical utility (eg, decision curve analysis). This process is referred to as evaluating a prediction model.^74^ ^75^

**Outcome**

Diagnostic or prognostic event that is being predicted. In machine learning, this event is often referred to as the target value, response variable, or label.

**Predictor**

Characteristic that can be measured or attributed at an individual level (eg, age, systolic blood pressure, sex, disease stage, radiomics features) or group level (eg, country). It is also often referred to as an input, feature, independent variable, or covariate.

**Training or development data**

Data used to train or develop a prediction model. The training data are ideally representative of the population in whom the model is to be used.

*TRIPOD=Transparent Reporting of a multivariable prediction model for Individual Prognosis Or Diagnosis; AI=artificial intelligence.†Validation data often has different meanings. For example, in machine learning studies, validation data can refer to data used for parameter tuning or data used to evaluate model performance (often referred to as external validation). To avoid any ambiguity, we refer to data used to evaluate model performance as evaluation data.

**Summary points**

There has been considerable interest and financial investment in developing prediction models by applying artificial intelligence (AI) methods, typically powered by advances in machine learning
To ensure that a prediction model study is valuable to users, authors should prepare a transparent, complete, and accurate account of why the research was done, what they did, and what they found
An update of the TRIPOD (Transparent Reporting of a multivariable prediction model for Individual Prognosis Or Diagnosis) statement aims to harmonise the landscape of prediction model studies using AI methods and to provide guidance regardless of whether regression models or machine learning methods have been used
The TRIPOD+AI statement consists of a 27 item checklist, an expanded checklist that details reporting recommendations for each item, and a TRIPOD+AI for Abstracts checklist containing 13 items
TRIPOD+AI aims to assist authors in the complete reporting of their study and help peer reviewers, editors, policymakers, end users, and patients understand the data, methods, findings and conclusions of AI driven research
Adherence to the TRIPOD+AI reporting recommendations could encourage the improved use of research time, effort, and money

## Development of TRIPOD+AI

We describe the development of the TRIPOD+AI statement, a guideline to aid the reporting of studies developing prediction models for diagnosis or prognosis using machine learning or regression methods or evaluating (validating) their performance. There is no such thing as a validated prediction model.^76^ To avoid ambiguity and harmonise terminology, we refer to validation as evaluation^74^ in this article (box 1). Existing reporting guidelines and those in development for reporting other types of biomedical studies involving a machine learning component are detailed in table 1. Literature reviews and consensus exercises were used to develop the TRIPOD+AI checklist as recommended by the EQUATOR Network.^77^ A steering group was convened by GSC and KGMM to oversee the guideline development process, with members selected to cover a broad range of expertise and experience (comprising GSC, KGMM, RDR, ALB, JBR, BVC, XL, and PD).

In April 2019, a commentary was published announcing the TRIPOD+AI initiative.^78^ The guideline was registered as a reporting guideline under development with the EQUATOR Network on 7 May 2019 (https://www.equator-network.org/). A study protocol was made available on 25 March 2021 on the Open Science Framework (https://osf.io/zyacb/), describing the process and methods used to develop the TRIPOD+AI reporting guideline. The protocol, which also describes the development of a quality assessment and risk-of-bias tool for prediction models developed using machine learning methods (PROBAST+AI), was published in 2021.^79^ The reporting of the consensus based methods used in the development of TRIPOD+AI followed the ACCORD (Accurate Consensus Reporting Document) recommendations.^80^

### Ethics

This study was approved by the Central University research ethics committee, University of Oxford on 10 December 2020 (R73034/RE001). Participant information was provided to the Delphi survey participants electronically before starting the survey and to the consensus participants before the consensus meeting. Delphi survey participants provided electronic informed consent before completing the survey.

### Candidate item list generation

An initial list of items was drafted by GSC and KGMM using TRIPOD 2015.^16^ ^17^ Additional items were identified from TRIPOD-Cluster,^19^ ^20^ TRIPOD for Abstracts,^18^ CAIR,^81^ MI-CLAIM,^82^ CLAIM,^68^ MINIMAR,^83^ SPIRIT-AI,^71^ and CONSORT-AI,^72^ along with additional literature identified by the steering group.^34^ ^84^ ^85^ ^86^ ^87^ ^88^ ^89^ The list of items was also informed by the findings of systematic reviews evaluating the reporting, methods, and overinterpretation of prediction model studies using machine learning.^37^ ^38^ ^39^ ^48^ ^51^ ^54^ ^59^ ^60^ The steering group harmonised the initial list of items to form a final list of 65 unique candidate items covering the title (one item); abstract (one item); introduction (three items); methods (37 items); results (15 items); discussion (five items), and other (three items). This list was used in a modified Delphi exercise as described below.

### Recruitment of Delphi panellists

Delphi participants were identified by the steering committee, from authors of relevant publications via a call to participate on social media (eg, Twitter), and through personal recommendations. Including experts recommended by other Delphi participants. The steering group identified participants to achieve geographical and disciplinary diversity and include key stakeholder groups, for example, researchers (statisticians/data scientists, epidemiologists, machine learning researchers/scientists, clinicians, radiologists, and ethicists), healthcare professionals, journal editors, funders, policymakers, healthcare regulators, patients, and the general public as end users of prediction models from a range of settings (eg, universities, hospitals, primary care, biomedical journals, non-profit organisations, and for-profit organisations). 

No minimum sample size was placed on the number of Delphi participants. A steering group member checked the expertise or experience of each identified person. Individuals were then invited to participate via email and were sent an information pack with the study description, aims, and contact details. Once participants accepted, they were added to the Delphi panel and received the link to the survey. Delphi panellists did not receive any financial incentive or gift to participate.

### Delphi process

The Delphi surveys were designed and delivered electronically using the Welphi online platform (www.welphi.com) to be responded to individually, online, and in English. The platform ensures responses are anonymous by sending a different link to each participant and applying codes to respondents. The panellists received a package of information clarifying the study’s objectives and scope and explaining how to participate, use the platform, and contact the development team with any questions. Participants were asked to rate each item as “can be omitted,” “possibly include,” “desirable for inclusion,” or “essential for inclusion.” Participants were also invited to comment on any item, and to suggest new items. Free text responses were collated and analysed by PL. The themes generated were used by GSC and KGMM to inform item rephrasing, merging, or suggesting new items. All members of the steering group were invited to participate in the Delphi surveys.

### Round 1 participants

The invitation and participation link was sent to 292 people. The first round was conducted between 19 April 2021 and 13 May 2021. A reminder message was sent on 5 May 2021. Of 292 people invited, 170 completed the survey, including eight who provided partial responses. Survey participants came from 22 countries, predominantly the UK (n=52), US (n=31), Netherlands (n=23), and Canada (n=20), representing five continents (Europe: 100, South America: 2, North America: 51, Australasia: 4, Asia: 13). Seven participants did not declare their country.

Participants reported their primary fields of research/work and could select more than one field. They indicated statistics and data science (n=70), AI or machine learning (n=69), clinical (n=50) or epidemiology (n=40), prediction (n=18), radiology (n=18), health policy/regulatory (n=10), biomedical research (n=7), journal editor (n=6), meta-research/reporting (n=6), pathology (n=2), funder (n=2), ethics (n=2), technology development/implementation (n=2), genetics/genomics (n=2), biomedical engineering (n=2), and health economics (n=2).

### Round 2 participants

The second round of the Delphi was conducted between 16 December 2021 and 17 January 2022. All participants who completed the first Delphi round were invited to the second Delphi round. Additional participants who did not respond to round 1 were reinvited, as were participants identified or recommended after round 1. Invitations for round 2 were sent to 395 people, of whom 200 completed the survey, including 15 who provided partial responses. Survey participants came from 27 different countries, again predominantly the UK (n=70), US (n=37), Netherlands (n=19), and Canada (n=19), and represented six continents (Europe: 123, South America: 3, North America: 56, Australasia: 7, Asia: 10, Africa: 1). Participants reported their primary fields of research/work and could select more than one field: statistics and data science (n=78), AI or machine learning (n=72), clinical (n=49) or epidemiology (n=51), prediction (n=19), radiology (n=26), health policy (n=12), biomedical research (n=14), journal editor (n=13), meta-research/reporting (n=6), biomedical engineering (n=5), funder (n=2), genetics/genomics (n=4), patient representative/engagement (n=3), health economics (n=2), and ethics (n=1).

### Checklist item evolution from round 1 to round 2

In round 1 of the modified Delphi, participants rated 65 initial candidate items generated from literature reviews and other reporting checklists, as described above. Agreement was considered when the individuals agreed an item was desirable or essential for inclusion. As defined in the protocol,^79^ items with agreement of 70% or higher were carried over to round 2. Items that had an agreement rate lower than 70% were excluded, merged, or rephrased to be presented to panellists for revaluation. These modifications were based on or inspired by the hundreds of comments added by panellists.

In round 2, survey participants were given a link to the aggregated ratings from round 1 (https://osf.io/zyacb/; supplementary table 3). In round 2, participants rated 59 candidate items, covering the title (one item), abstract (one item), introduction (four items), methods (32 items), results (11 items), discussion (eight items), and other (two items). The item relating to patient and public involvement received 69% agreement for inclusion (supplementary table 4). Despite falling just below the 70% threshold, the steering group agreed to retain this item for discussion during the consensus meeting.

### Patient and public involvement and engagement meeting

An online meeting was held on 8 April 2022 with nine members of the Health Data Research UK’s group for patient and public involvement and engagement (PPIE) (https://www.hdruk.ac.uk/about-us/involving-and-engaging-patients-and-the-public/), chaired by Sophie Staniszewska **(**University of Warwick, UK). This meeting was not planned in the study protocol and was the only deviation from the published protocol.^79^ Before the meeting, the PPIE group was sent a summary of the TRIPOD+AI project (available at https://osf.io/zyacb/), including an executive summary drafted by one member of PPIE group, and the draft checklist. At the meeting, GSC presented details of the TRIPOD+AI initiative, the project status, and draft guidance resulting from round 2 of the Delphi survey. Participants then asked questions and discussed the project aims and scope. Following feedback received at the PPI meeting, and through correspondence written after the meeting, the draft checklist was revised to improve clarity. Three members of the PPI group were invited and two subsequently attended the online consensus meeting with the wider group of stakeholders on 5 July 2022. The manuscript was circulated to the three PPI members for their input and approval.

### Consensus meeting

An online consensus meeting was held on 5 July 2022, chaired by GSC and KGMM. Participants were chosen to try to ensure balanced representation of the key stakeholder groups, disciplines, and geographical diversity. Twenty eight participants attended part or all of the meeting, including one non-voting attendee (PL).

Before the meeting, invited participants were emailed a document (available at https://osf.io/zyacb/) containing a brief overview of TRIPOD+AI, the consensus meeting format and instructions, a summary of the aggregated responses from round 2 of the Delphi survey (supplementary table 3), and the draft TRIPOD+AI checklist. The checklist circulated to the consensus meeting participants included 59 items covering: title (one item), abstract (one item), introduction (four items), methods (32 items), results (11 items), discussion (eight items), and other (two items).

Given the high endorsement achieved for many items in round 2, a subset of 17 items were highlighted for plenary discussion and voting during the consensus meeting. After discussion, participants were given one minute to vote to include or exclude the item from the TRIPOD+AI checklist. The voting was registered using the poll function of the online meeting program. The 17 items included one item that had not achieved consensus in round 2 and 16 items that had undergone rewording after round 2 or were new items that were not included in TRIPOD 2015. After discussion and voting on these 17 items, the final TRIPOD+AI checklist was formed.

## TRIPOD+AI statement

TRIPOD+AI comprises a checklist of items that are considered essential for good reporting of studies developing or evaluating (validating) a prediction model using any statistical or machine learning methods (table 2). Box 2 summarises noteworthy additions and changes to TRIPOD 2015. The TRIPOD+AI checklist comprises 27 main items about the title (item 1), abstract (item 2), introduction (items 3 and 4), methods (items 5-17), open science practises (item 18), patient and public involvement (item 19), results (items 20-24), and discussion (items 25-27). Some items included multiple subitems, totalling to 52 checklist subitems.

**Table 2:** TRIPOD+AI checklist for the reporting of prediction model studies

Section/topic	Item	Development/evaluation*	Checklist item
**Title**			
Title	1	D;E	Identify the study as developing or evaluating the performance of a multivariable prediction model, the target population, and the outcome to be predicted
**Abstract**			
Abstract	2	D;E	See TRIPOD+AI for Abstracts checklist
**Introduction**			
Background	3a	D;E	Explain the healthcare context (including whether diagnostic or prognostic) and rationale for developing or evaluating the prediction model, including references to existing models
	3b	D;E	Describe the target population and the intended purpose of the prediction model in the context of the care pathway, including its intended users (eg, healthcare professionals, patients, public)
	3c	D;E	Describe any known health inequalities between sociodemographic groups
Objectives	4	D;E	Specify the study objectives, including whether the study describes the development or validation of a prediction model (or both)
**Methods**			
Data	5a	D;E	Describe the sources of data separately for the development and evaluation datasets (eg, randomised trial, cohort, routine care or registry data), the rationale for using these data, and representativeness of the data
	5b	D;E	Specify the dates of the collected participant data, including start and end of participant accrual; and, if applicable, end of follow-up
Participants	6a	D;E	Specify key elements of the study setting (eg, primary care, secondary care, general population) including the number and location of centres
	6b	D;E	Describe the eligibility criteria for study participants
	6c	D;E	Give details of any treatments received, and how they were handled during model development or evaluation, if relevant
Data preparation	7	D;E	Describe any data pre-processing and quality checking, including whether this was similar across relevant sociodemographic groups
Outcome	8a	D;E	Clearly define the outcome that is being predicted and the time horizon, including how and when assessed, the rationale for choosing this outcome, and whether the method of outcome assessment is consistent across sociodemographic groups
	8b	D;E	If outcome assessment requires subjective interpretation, describe the qualifications and demographic characteristics of the outcome assessors
	8c	D;E	Report any actions to blind assessment of the outcome to be predicted
Predictors	9a	D	Describe the choice of initial predictors (eg, literature, previous models, all available predictors) and any pre-selection of predictors before model building
	9b	D;E	Clearly define all predictors, including how and when they were measured (and any actions to blind assessment of predictors for the outcome and other predictors)
	9c	D;E	If predictor measurement requires subjective interpretation, describe the qualifications and demographic characteristics of the predictor assessors
Sample size	10	D;E	Explain how the study size was arrived at (separately for development and evaluation), and justify that the study size was sufficient to answer the research question. Include details of any sample size calculation
Missing data	11	D;E	Describe how missing data were handled. Provide reasons for omitting any data
Analytical methods	12a	D	Describe how the data were used (eg, for development and evaluation of model performance) in the analysis, including whether the data were partitioned, considering any sample size requirements
	12b	D	Depending on the type of model, describe how predictors were handled in the analyses (functional form, rescaling, transformation, or any standardisation)
	12c	D	Specify the type of model, rationale†, all model building steps, including any hyperparameter tuning, and method for internal validation
	12d	D;E	Describe if and how any heterogeneity in estimates of model parameter values and model performance was handled and quantified across clusters (eg, hospitals, countries). See TRIPOD-Cluster for additional considerations‡
	12e	D;E	Specify all measures and plots used (and their rationale) to evaluate model performance (eg, discrimination, calibration, clinical utility) and, if relevant, to compare multiple models
	12f	E	Describe any model updating (eg, recalibration) arising from the model evaluation, either overall or for particular sociodemographic groups or settings
	12g	E	For model evaluation, describe how the model predictions were calculated (eg, formula, code, object, application programming interface)
Class imbalance	13	D;E	If class imbalance methods were used, state why and how this was done, and any subsequent methods to recalibrate the model or the model predictions
Fairness	14	D;E	Describe any approaches that were used to address model fairness and their rationale
Model output	15	D	Specify the output of the prediction model (eg, probabilities, classification). Provide details and rationale for any classification and how the thresholds were identified
Training versus evaluation	16	D;E	Identify any differences between the development and evaluation data in healthcare setting, eligibility criteria, outcome, and predictors
Ethical approval	17	D;E	Name the institutional research board or ethics committee that approved the study and describe the participant informed consent or the ethics committee waiver of informed consent
**Open science**			
Funding	18a	D;E	Give the source of funding and the role of the funders for the present study
Conflicts of interest	18b	D;E	Declare any conflicts of interest and financial disclosures for all authors
Protocol	18c	D;E	Indicate where the study protocol can be accessed or state that a protocol was not prepared
Registration	18d	D;E	Provide registration information for the study, including register name and registration number, or state that the study was not registered
Data sharing	18e	D;E	Provide details of the availability of the study data
Code sharing	18f	D;E	Provide details of the availability of the analytical code§
**Patient and public involvement**			
Patient and public involvement	19	D;E	Provide details of any patient and public involvement during the design, conduct, reporting, interpretation, or dissemination of the study or state no involvement
**Result**			
Participants	20a	D;E	Describe the flow of participants through the study, including the number of participants with and without the outcome and, if applicable, a summary of the follow-up time. A diagram may be helpful
	20b	D;E	Report the characteristics overall and, where applicable, for each data source or setting, including the key dates, key predictors (including demographics), treatments received, sample size, number of outcome events, follow-up time, and amount of missing data. A table may be helpful. Report any differences across key demographic groups
	20c	E	For model evaluation, show a comparison with the development data of the distribution of important predictors (demographics, predictors, and outcome)
Model development	21	D;E	Specify the number of participants and outcome events in each analysis (eg, for model development, hyperparameter tuning, model evaluation)
Model specification	22	D	Provide details of the full prediction model (eg, formula, code, object, application programming interface) to allow predictions in new individuals and to enable third party evaluation and implementation, including any restrictions to access or reuse (eg, freely available, proprietary)¶
Model performance	23a	D;E	Report model performance estimates with confidence intervals, including for any key subgroups (eg, sociodemographic). Consider plots to aid presentation
	23b	D;E	If examined, report results of any heterogeneity in model performance across clusters. See TRIPOD-Cluster for additional details‡
Model updating	24	E	Report the results from any model updating, including the updated model and subsequent performance
**Discussion**			
Interpretation	25	D;E	Give an overall interpretation of the main results, including issues of fairness in the context of the objectives and previous studies
Limitations	26	D;E	Discuss any limitations of the study (such as a non-representative sample, sample size, overfitting, missing data) and their effects on any biases, statistical uncertainty, and generalisability
Usability of the model in the context of current care	27a	D	Describe how poor quality or unavailable input data (eg, predictor values) should be assessed and handled when implementing the prediction model
	27b	D	Specify whether users will be required to interact in the handling of the input data or use of the model, and what level of expertise is required of users
	27c	D;E	Discuss any next steps for future research, with a specific view to applicability and generalisability of the model

TRIPOD=Transparent Reporting of a multivariable prediction model for Individual Prognosis Or Diagnosis; AI=artificial intelligence.

* D=items relevant only to the development of a prediction model; E=items relating solely to the evaluation of a prediction model; D;E=items applicable to both the development and evaluation of a prediction model.

† Separately for all model building approaches.

‡ TRIPOD-Cluster is a checklist of reporting recommendations for studies developing or validating models that explicitly account for clustering or explore heterogeneity in model performance (eg, at different hospitals or centres).^19^ ^20^

§ Relates to the analysis code, for example, any data cleaning, feature engineering, model building, and evaluation.

¶ Relates to the code to implement the model to get estimates of risk for a new individual.

Box 2 **Noteworthy changes and additions to TRIPOD 2015**

- New checklist of reporting recommendations to cover prediction model studies using any regression or machine learning method (eg, random forests, deep learning), and harmonise nomenclature between regression and machine learning communities
- New TRIPOD+AI checklist supersedes the TRIPOD 2015 checklist, which should no longer be used
- Particular emphasis on fairness (box 1) to raise awareness and ensure that reports mention whether specific methods were used to deal with fairness. Aspects of fairness are embedded throughout the checklist
- Inclusion of TRIPOD+AI for Abstracts for guidance on reporting abstracts
- Modification of the model performance item recommending that authors evaluate model performance in key subgroups (eg, sociodemographic)
- Inclusion of a new item on patient and public involvement to raise awareness and prompt authors to provide details on any patient and public involvement during the design, conduct, reporting (and interpretation), and dissemination of the study
- Inclusion of an open science section with subitems on study protocols, registration, data sharing and code sharing

TRIPOD=Transparent Reporting of a multivariable prediction model for Individual Prognosis or Diagnosis; AI=artificial intelligence.

TRIPOD+AI covers studies that describe the development of a prediction model, the evaluation (validation) of prediction model performance, or both. Any items denoted D;E apply to all studies regardless of whether they are developing a prediction model or evaluating the performance of a prediction model (table 2). Items in the checklist denoted D apply to studies that describe the development of a prediction model, while items denoted E apply to studies that evaluate the performance of a prediction model. For studies both developing and evaluating the performance of a prediction model, all checklist items apply.

A separate checklist for journal or conference abstracts of prediction model studies is included in TRIPOD+AI. This checklist updates the TRIPOD for Abstracts statement,^18^ reflecting new content and maintaining consistency with TRIPOD+AI (table 3).

**Table 3:** Essential items to include for the reporting of prediction model studies in a journal or conference abstract (TRIPOD+AI for Abstracts*)

Section and item	Checklist item
**Title**	
1	Identify the study as developing or evaluating the performance of a multivariable prediction model, the target population, and the outcome to be predicted
**Background**	
2	Provide a brief explanation of the healthcare context and rationale for developing or evaluating the performance of all models
**Objectives**	
3	Specify the study objectives, including whether the study describes model development, evaluation, or both
**Methods**	
4	Describe the sources of data
5	Describe the eligibility criteria and setting where the data were collected
6	Specify the outcome to be predicted by the model, including time horizon of predictions in case of prognostic models
7	Specify the type of model, a summary of the model-building steps, and the method for internal validation†
8	Specify the measures used to assess model performance (eg, discrimination, calibration, clinical utility)
**Results**	
9	Report the number of participants and outcome events
10	Summarise the predictors in the final model†
11	Report model performance estimates (with confidence intervals)
**Discussion**	
12	Give an overall interpretation of the main results
**Registration**	
13	Give the registration number and name of the registry or repository

TRIPOD=Transparent Reporting of a multivariable prediction model for Individual Prognosis Or Diagnosis; AI=artificial intelligence.

* This checklist is based on the TRIPOD for Abstracts statement published in 2020,^17^ but has been revised and updated for consistency with the TRIPOD+AI statement.

† Relevant only to studies describing the development of a prediction model.

The recommendations in TRIPOD+AI are for transparently reporting how prediction model research was conducted; it does not prescribe how to develop or evaluate a prediction model. The checklist is not a quality appraisal tool. Readers are referred to PROBAST^90^ ^91^ and the forthcoming PROBAST+AI^79^ to assess the quality and risk of bias of prediction models (https://www.probast.org/).

## How to use TRIPOD+AI

The TRIPOD+AI checklist supersedes the TRIPOD 2015 checklist, which should no longer be used. For prediction model studies that have accounted for clustering (eg, multiple hospitals, multiple datasets), authors should consult TRIPOD-Cluster for additional reporting recommendations.^19^ ^20^ The 2015 explanation and elaboration document remains an important document to provide background and examples for most of the TRIPOD+AI reporting items^17^ (because many items have not changed or have been minimally changed), while we produce a detailed and updated document for TRIPOD+AI. We recommend using TRIPOD+AI early in the writing process to ensure that all key details are addressed and reported. An expanded checklist in a bullet point structure has been developed (supplementary table 1) to facilitate implementation of TRIPOD+AI by providing a brief rationale and guidance for each item in the checklist.

Although many of the items in the TRIPOD+AI checklist have a natural order and sequence in a report, some do not. We do not stipulate a structured format or dictate where each individual reporting recommendation should appear in a prediction model report or publication, because this order might also depend on journal formatting policies.

The recommendations contained within TRIPOD+AI are the minimum reporting recommendations, and authors may provide additional information. If journal word limits and restrictions on number of tables and figures in the main body of the manuscript complicate reporting, authors can report and reference some of the requested or additional information in supplementary material. If the information required is already reported in a publicly accessible study protocol, then referring to that document may suffice. If a particular checklist item cannot be discussed in the report because the information is unknown or irrelevant, then this should be acknowledged and clearly stated. Additional files and study materials not included in the supplementary material should be deposited in general purpose (eg, Open Science Framework, Dryad, figshare) or institutional open access repositories that provide free access in perpetuity. Details of access to any additional files should be referenced and linked, for example, with a doi number in the main study report or publication.

We recommend that authors submit a completed checklist indicating the page or line where each requested item can be found, to help the editorial and peer review process. A template for the TRIPOD+AI checklist for filling out separately can be found in supplementary table 2 and is available to download from www.tripod-statement.org.

News, announcements, and information relating to TRIPOD+AI can be found on the TRIPOD website (www.tripod-statement.org) and on social media accounts such as X (formerly known as Twitter; @TRIPODStatement). The Enhancing the Quality and Transparency Of health Research (EQUATOR) Network (https://www.equator-network.org/) will also disseminate and promote the TRIPOD+AI statement. Translation of TRIPOD+AI into different languages is welcomed and encouraged, please contact the corresponding author. Translations should use the structured and predefined process that includes authors of the original publication and receives their approval. The TRIPOD website contains further details on translation (www.tripod-statement.org).

## Discussion

TRIPOD+AI has been developed through an international multi-stakeholder consensus process. It provides minimum reporting recommendations for studies describing the development or evaluation (validation) of prediction models using any regression or machine learning methods. At the time of guideline development, foundation and large language models (such as ChatGPT) that are rapidly gaining momentum were not considered—the TRIPOD+AI guidance is primarily aimed at non-generative models. However, many of the principles are applicable for driving transparency in generative AI studies in health. Periodic updating of TRIPOD+AI will be needed to remain relevant and reflect advancements in AI and machine learning methods, for example, by explicitly looking at generative approaches.

TRIPOD+AI was developed by updating TRIPOD 2015, with recommendations informed by systematic reviews of the literature, a Delphi survey, and an online consensus meeting. Reporting TRIPOD+AI items can help users to understand and appraise the quality of the study methods, increasing transparency around the study findings, reducing overinterpretation of study findings, facilitating replication and reproducibility, and aiding implementation of the prediction model. The checklist items are minimum reporting recommendations, and authors will typically provide additional details on the data, study design, methods, analysis, results, and discussion.

TRIPOD+AI emphasises fairness issues throughout the checklist, which was lacking or not explicitly stated in TRIPOD 2015.^33^ Fairness in prediction model research is particularly important in healthcare, which has gained prominence with AI and machine learning methods being used to develop models to assist in decision making. Fairness in this context means that prediction models are designed and used in a way that does not adversely discriminate against any particular group of individuals and does not create or exacerbate (and ideally mitigates or reduces) existing inequalities in healthcare provision or patient outcomes.^92^ One important aspect of fairness is ensuring that the data used to develop or evaluate prediction models are representative and diverse, and that limitations of data bias are acknowledged, dealt with, and mitigated during model development. The STANDING Together initiative is in the process of developing standards for data diversity, inclusivity, and generalisability to tackle bias in AI health datasets.^62^

Data should ideally include information from individuals of different ages, sexes/genders, and races/ethnicities, with different health conditions or comorbidities and from different geographical locations. These differences should be representative of the population in whom the prediction model is intended to be used. If the data used to develop the models do not adequately represent the full diversity of the intended use population, the resulting model might not perform as expected in those missing from the data, which should be clearly stated. If the data used to evaluate a model are not representative of the target population, then the estimated predictive accuracy in subgroups (eg, defined by relevant personal, social, or clinical attributes) could be biased and misleading.

While adequate representation of minoritised and underserved groups within datasets is one key element to achieving fairness goals, representation alone does not guarantee fairness.^61^ ^93^ Therefore, TRIPOD+AI has embedded items on fairness throughout, including in the background (item 3c), methods (items 5a, 7, 8a, 8b, 9c, 12f, 14), results (items 20b, 23a), and discussion (items 25, 26).

Fairness in healthcare also means involving diverse stakeholders, including patients, the general public, and clinicians, in the development, evaluation, implementation, and deployment of a prediction model into the clinical pathway.^94^ Involving a variety of perspectives will help to ensure that the prediction model is, in principle, designed to meet the needs of all individuals and is used in a way that is fair and equitable, promoting health equity. TRIPOD+AI includes item 19 on public and patient involvement to incentivise the integration of patient involvement in prediction model studies beyond a mere tick box exercise, to encourage and promote the principles of open science and engagement, and to ensure better clinical and public acceptability of the work.

TRIPOD+AI prominently features open science practices.^35^ Open science practices are crucial for prediction model research in healthcare as they promote transparency, reproducibility, and collaboration between researchers.^95^ By registering research and making study materials such as protocols, data, code, and the prediction model open available, other researchers can verify the findings and evaluate model performance in new data to ensure that models are accurate, and evaluate models for safety. Open science practices also enable researchers to build on each other’s work, leading to more efficient progress in healthcare. These practices can have a considerable impact on patient outcomes by improving the accuracy, integrity, and reliability of prediction models. If data are openly shared, clinicians and researchers can develop or evaluate models on larger and more diverse sets of patient data,^96^ potentially leading to more accurate predictions and better informed decisions for patient healthcare. Therefore, TRIPOD+AI includes a section on open science, covering issues such as funding declarations (item 18a), conflicts of interest (18b), protocol availability (18c), study registration (18d), data sharing (18e), and code sharing (18f).

We anticipate that the key users and beneficiaries of TRIPOD+AI will be researchers writing papers, journal editors and peer reviewers who evaluate research papers, and other stakeholders (eg, academic institutions, policy makers, funders, regulators, patients, study participants, and the broader public) who will benefit from the increased quality of prediction model research (table 4). The guideline is relevant for any reports related to clinical prediction model development and validation studies, including medical research articles and other areas where evidenced reports are needed, for example, to accompany software and tools.

**Table 4:** Adherence to the TRIPOD+AI reporting guideline: potential benefits from stakeholders’ actions

User/stakeholder	Proposed action	Potential benefits
Academic institutions	Promote or require adherence of TRIPOD+AI by investigators developing, evaluating, or implementing prediction models	Enhance a culture of transparency in the design, analysis, and reporting of prediction model research
	Provide training for early career researchers on the importance and benefits of transparent and complete reporting, including requiring doctoral students to write their thesis and manuscripts in accordance with the full TRIPOD+AI guideline	Improve the quality, accountability, reproducibility, replicability, and usefulness of produced research
Researchers	Adhere to TRIPOD+AI when writing studies for publication	Improved completeness and quality of reporting
		Increased awareness of the minimal detail required and expected when writing a prediction model publication
		Improved quality, accountability, reproducibility, replicability, and usefulness of produced research
		Improved reporting of details that facilitate independent evaluation of the model
Journal editors	Require and enforce authors to use TRIPOD+AI and submit a completed a checklist when writing the manuscript	Improved understanding of journal requirements and expectations for prediction model publications
	Recommend peer reviewers use TRIPOD+AI	Increased efficiency of peer review resulting from improved author understanding of journal requirements for prediction model publications
		Improved quality, accountability, reproducibility, replicability, and usefulness of published research
Peer reviewers	Use TRIPOD+AI to evaluate completeness of reporting	Improve the efficiency and quality of peer review
		Facilitate and direct specific feedback to authors on where important details are missing
Funders	Recommend or mandate use of TRIPOD+AI by investigators when receiving a grant for prediction model research	Increase the usefulness of research outputs
		Reduce avoidable research waste due to incomplete reporting
		Ensure that funded research can be used by others
Patients, public, and study participants	Advocate use of TRIPOD+AI by authors, peer reviewers, journals, and funders	Improved trust in research findings
		Improved understanding of prediction model research
		Promote health equity considerations in research
		Align patient reported outcomes and patient experience with clinical research outcomes for precision medicine and personalised disease management
Systematic reviewers and meta-researchers	Use TRIPOD+AI to assess completeness of reporting	Improved evaluation of study quality when used alongside risk of bias tools (eg, PROBAST)
	Use TRIPOD+AI as an aid when assessing quality and risks of bias	Improved availability of data needed for meta-analysis
Policy makers	Use or promote TRIPOD+AI to ensure research is transparently and completely reported	Ensure decisions to evaluate or implement a prediction model are based on complete and transparently reported information
		Add integrity for evidence based policy recommendations
Regulators	Clinical reviewers use TRIPOD+AI to assess completeness of clinical investigation reporting for “software as a medical device” regulatory submissions where the operating principle of the product is a prediction model	Align reported intended use with regulatory intended purpose
		Align medical device regulatory review and pivotal investigational reporting with best practice
		Encourage manufacturers to publish clinical investigation reports by encouraging one common standard
Technology and medical device manufacturers	Verify whether sufficient details about a model are available to enable development and manufacturing of technology and devices	Encourages manufacturers to publish clinical investigation reports by encouraging one common standard
Healthcare professionals	Verify whether sufficient details about a model are available before purchasing or using a model to support clinical use	Improved understanding of the target population of a model and the clinical decision it is intended to support
		Improved understanding of model predictions and awareness of limitations
		Improved trust in research findings

TRIPOD=Transparent Reporting of a multivariable prediction model for Individual Prognosis Or Diagnosis; AI=artificial intelligence.

We encourage editors and publishers to support adherence to TRIPOD+AI by referring to it in journals’ instructions to authors, enforcing its use during the submission and peer review process, and making adherence to the recommendations an expectation. We also encourage funders to require that funding applications for prediction model studies include a plan to report their prediction model according to the TRIPOD+AI recommendations, thereby minimising research waste and ensuring value for money.

## Data Availability

Aggregated Delphi survey responses are available on the Open Science Framework TRIPOD+AI repository https://osf.io/zyacb/.

## Web extra

Extra material supplied by authors

Supplementary table 1: TRIPOD+AI Expanded Checklist (Explanation and Elaboration Light)

Supplementary table 2: Fillable TRIPOD+AI checklist

Supplementary table 3: Aggregated TRIPOD+AI responses from Delphi round 1

Supplementary table 4: Aggregated TRIPOD+AI responses from Delphi round 2

## Notes

Contributors: GSC and KGMM conceived the study and this paper and are joint first authors. GSC, PL, PD, RDR, ALB, BVC, XL, JBR, MvS, and KGMM designed the surveys carried out to inform the guideline content. PL analysed the survey results and free text comments from the surveys. GSC designed the materials for the consensus meeting with input from KGMM. All authors except SR, MMH, XL, SD, BG, and ALB attended the consensus meeting. PL took consolidated notes from the consensus meeting. GSC drafted the manuscript with input and edits from KGMM. All authors were involved in revising the article critically for important intellectual content and approved the final version of the article. GSC is the guarantor of this work. The corresponding author attests that all listed authors meet authorship criteria and that no others meeting the criteria have been omitted.

Provenance and peer review: Not commissioned; externally peer reviewed.

## References

[1] van Smeden M Reitsma JB Riley RD Collins GS Moons KG . Clinical prediction models: diagnosis versus prognosis. J Clin Epidemiol 2021;132:142-5. 10.1016/j.jclinepi.2021.01.009 33775387

[2] Nashef SA Roques F Sharples LD . EuroSCORE II. Eur J Cardiothorac Surg 2012;41:734-45. 10.1093/ejcts/ezs043 22378855

[3] Gail MH Brinton LA Byar DP . Projecting individualized probabilities of developing breast cancer for white females who are being examined annually. J Natl Cancer Inst 1989;81:1879-86. 10.1093/jnci/81.24.1879 2593165

[4] D’Agostino RB Sr Vasan RS Pencina MJ . General cardiovascular risk profile for use in primary care: the Framingham Heart Study. Circulation 2008;117:743-53. 10.1161/CIRCULATIONAHA.107.699579 18212285

[5] Steyerberg EW Mushkudiani N Perel P . Predicting outcome after traumatic brain injury: development and international validation of prognostic scores based on admission characteristics. PLoS Med 2008;5:e165. 10.1371/journal.pmed.0050165 18684008 PMC2494563

[6] Kanis JA Oden A Johnell O . The use of clinical risk factors enhances the performance of BMD in the prediction of hip and osteoporotic fractures in men and women. Osteoporos Int 2007;18:1033-46. 10.1007/s00198-007-0343-y 17323110

[7] Damen JAAG Hooft L Schuit E . Prediction models for cardiovascular disease risk in the general population: systematic review. BMJ 2016;353:i2416. 10.1136/bmj.i2416 27184143 PMC4868251

[8] Bellou V Belbasis L Konstantinidis AK Tzoulaki I Evangelou E . Prognostic models for outcome prediction in patients with chronic obstructive pulmonary disease: systematic review and critical appraisal. BMJ 2019;367:l5358. 10.1136/bmj.l5358 31585960 PMC6776831

[9] Wynants L Van Calster B Collins GS . Prediction models for diagnosis and prognosis of covid-19: systematic review and critical appraisal. BMJ 2020;369:m1328. 10.1136/bmj.m1328 32265220 PMC7222643

[10] Mallett S Royston P Dutton S Waters R Altman DG . Reporting methods in studies developing prognostic models in cancer: a review. BMC Med 2010;8:20. 10.1186/1741-7015-8-20 20353578 PMC2856521

[11] Collins GS Mallett S Omar O Yu LM . Developing risk prediction models for type 2 diabetes: a systematic review of methodology and reporting. BMC Med 2011;9:103. 10.1186/1741-7015-9-103 21902820 PMC3180398

[12] Altman DG Simera I Hoey J Moher D Schulz K . EQUATOR: reporting guidelines for health research. Open Med 2008;2:e49-50. 21602941 PMC3090180

[13] Glasziou P Altman DG Bossuyt P . Reducing waste from incomplete or unusable reports of biomedical research. Lancet 2014;383:267-76. 10.1016/S0140-6736(13)62228-X 24411647

[14] Collins GS de Groot JA Dutton S . External validation of multivariable prediction models: a systematic review of methodological conduct and reporting. BMC Med Res Methodol 2014;14:40. 10.1186/1471-2288-14-40 24645774 PMC3999945

[15] Bouwmeester W Zuithoff NP Mallett S . Reporting and methods in clinical prediction research: a systematic review. PLoS Med 2012;9:1-12. 10.1371/journal.pmed.1001221 22629234 PMC3358324

[16] Collins GS Reitsma JB Altman DG Moons KGM . Transparent Reporting of a multivariable prediction model for Individual Prognosis or Diagnosis (TRIPOD): the TRIPOD statement. Ann Intern Med 2015;162:55-63. 10.7326/M14-0697 25560714

[17] Moons KGM Altman DG Reitsma JB . Transparent Reporting of a multivariable prediction model for Individual Prognosis or Diagnosis (TRIPOD): explanation and elaboration. Ann Intern Med 2015;162:W1-73. 10.7326/M14-0698 25560730

[18] Heus P Reitsma JB Collins GS . Transparent Reporting of Multivariable Prediction Models in Journal and Conference Abstracts: TRIPOD for Abstracts. Ann Intern Med 2020;173:42-7. 10.7326/M20-0193 32479165

[19] Debray TPA Collins GS Riley RD . Transparent reporting of multivariable prediction models developed or validated using clustered data: TRIPOD-Cluster checklist. BMJ 2023;380:e071018. 10.1136/bmj-2022-071018 36750242 PMC9903175

[20] Debray TPA Collins GS Riley RD . Transparent reporting of multivariable prediction models developed or validated using clustered data (TRIPOD-Cluster): explanation and elaboration. BMJ 2023;380:e071058. 10.1136/bmj-2022-071058 36750236 PMC9903176

[21] Snell KIE Levis B Damen JAA . Transparent reporting of multivariable prediction models for individual prognosis or diagnosis: checklist for systematic reviews and meta-analyses (TRIPOD-SRMA). BMJ 2023;381:e073538. 10.1136/bmj-2022-073538 37137496 PMC10155050

[22] Dhiman P Whittle R Van Calster B . The TRIPOD-P reporting guideline for improving the integrity and transparency of predictive analytics in healthcare through study protocols. Nat Mach Intell 2023;5:816-7 10.1038/s42256-023-00705-6.

[23] Riley RD Snell KI Ensor J . Minimum sample size for developing a multivariable prediction model: PART II - binary and time-to-event outcomes. Stat Med 2019;38:1276-96. 10.1002/sim.7992 30357870 PMC6519266

[24] Riley RD Snell KIE Ensor J . Minimum sample size for developing a multivariable prediction model: Part I - Continuous outcomes. Stat Med 2019;38:1262-75. 10.1002/sim.7993 30347470

[25] Riley RD Ensor J Snell KIE . Calculating the sample size required for developing a clinical prediction model. BMJ 2020;368:m441. 10.1136/bmj.m441 32188600

[26] van Smeden M de Groot JA Moons KG . No rationale for 1 variable per 10 events criterion for binary logistic regression analysis. BMC Med Res Methodol 2016;16:163. 10.1186/s12874-016-0267-3 27881078 PMC5122171

[27] van Smeden M Moons KG de Groot JA . Sample size for binary logistic prediction models: Beyond events per variable criteria. Stat Methods Med Res 2019;28:2455-74. 10.1177/0962280218784726 29966490 PMC6710621

[28] Snell KIE Archer L Ensor J . External validation of clinical prediction models: simulation-based sample size calculations were more reliable than rules-of-thumb. J Clin Epidemiol 2021;135:79-89. 10.1016/j.jclinepi.2021.02.011 33596458 PMC8352630

[29] Archer L Snell KIE Ensor J Hudda MT Collins GS Riley RD . Minimum sample size for external validation of a clinical prediction model with a continuous outcome. Stat Med 2021;40:133-46. 10.1002/sim.8766 33150684

[30] Riley RD Debray TPA Collins GS . Minimum sample size for external validation of a clinical prediction model with a binary outcome. Stat Med 2021;40:4230-51. 10.1002/sim.9025 34031906

[31] Riley RD Collins GS Ensor J . Minimum sample size calculations for external validation of a clinical prediction model with a time-to-event outcome. Stat Med 2022;41:1280-95. 10.1002/sim.9275 34915593

[32] Riley RD Snell KIE Archer L . Evaluation of clinical prediction models (part 3): calculating the sample size required for an external validation study. BMJ 2024;384:e074821. 10.1136/bmj-2023-074821 38253388 PMC11778934

[33] Wawira Gichoya J McCoy LG Celi LA Ghassemi M . Equity in essence: a call for operationalising fairness in machine learning for healthcare. BMJ Health Care Inform 2021;28:e100289. 10.1136/bmjhci-2020-100289 33910923 PMC8733939

[34] McDermott MBA Wang S Marinsek N Ranganath R Foschini L Ghassemi M . Reproducibility in machine learning for health research: Still a ways to go. Sci Transl Med 2021;13:eabb1655. 10.1126/scitranslmed.abb1655 33762434 PMC12862614

[35] UNESCO. UNESCO Recommendation on Open Science. 2023. https://www.unesco.org/en/open-science/about?hub=686

[36] Wessler BS Nelson J Park JG . External Validations of Cardiovascular Clinical Prediction Models: A Large-Scale Review of the Literature. Circ Cardiovasc Qual Outcomes 2021;14:e007858. 10.1161/CIRCOUTCOMES.121.007858 34340529 PMC8366535

[37] Dhiman P Ma J Andaur Navarro CL . Methodological conduct of prognostic prediction models developed using machine learning in oncology: a systematic review. BMC Med Res Methodol 2022;22:101. 10.1186/s12874-022-01577-x 35395724 PMC8991704

[38] Andaur Navarro CL Damen JAA van Smeden M . Systematic review identifies the design and methodological conduct of studies on machine learning-based prediction models. J Clin Epidemiol 2023;154:8-22. 10.1016/j.jclinepi.2022.11.015 36436815

[39] Andaur Navarro CL Damen JAA Takada T . Completeness of reporting of clinical prediction models developed using supervised machine learning: a systematic review. BMC Med Res Methodol 2022;22:12. 10.1186/s12874-021-01469-6 35026997 PMC8759172

[40] Rech MM de Macedo Filho L White AJ . Machine Learning Models to Forecast Outcomes of Pituitary Surgery: A Systematic Review in Quality of Reporting and Current Evidence. Brain Sci 2023;13:495. 10.3390/brainsci13030495 36979305 PMC10046799

[41] Munguía-Realpozo P Etchegaray-Morales I Mendoza-Pinto C . Current state and completeness of reporting clinical prediction models using machine learning in systemic lupus erythematosus: A systematic review. Autoimmun Rev 2023;22:103294. 10.1016/j.autrev.2023.103294 36791873

[42] Kee OT Harun H Mustafa N . Cardiovascular complications in a diabetes prediction model using machine learning: a systematic review. Cardiovasc Diabetol 2023;22:13. 10.1186/s12933-023-01741-7 36658644 PMC9854013

[43] Song Z Yang Z Hou M Shi X . Machine learning in predicting cardiac surgery-associated acute kidney injury: A systemic review and meta-analysis. Front Cardiovasc Med 2022;9:951881. 10.3389/fcvm.2022.951881 36186995 PMC9520338

[44] Yang Q Fan X Cao X . Reporting and risk of bias of prediction models based on machine learning methods in preterm birth: A systematic review. Acta Obstet Gynecol Scand 2023;102:7-14. 10.1111/aogs.14475 36397723 PMC9780725

[45] Groot OQ Ogink PT Lans A . Machine learning prediction models in orthopedic surgery: A systematic review in transparent reporting. J Orthop Res 2022;40:475-83. 10.1002/jor.25036 33734466 PMC9290012

[46] Lans A Kanbier LN Bernstein DN . Social determinants of health in prognostic machine learning models for orthopaedic outcomes: A systematic review. J Eval Clin Pract 2023;29:292-9. 10.1111/jep.13765 36099267

[47] Li B Feridooni T Cuen-Ojeda C . Machine learning in vascular surgery: a systematic review and critical appraisal. NPJ Digit Med 2022;5:7. 10.1038/s41746-021-00552-y 35046493 PMC8770468

[48] Groot OQ Bindels BJJ Ogink PT . Availability and reporting quality of external validations of machine-learning prediction models with orthopedic surgical outcomes: a systematic review. Acta Orthop 2021;92:385-93. 10.1080/17453674.2021.1910448 33870837 PMC8436968

[49] Andaur Navarro CL Damen JAA Takada T . Risk of bias in studies on prediction models developed using supervised machine learning techniques: systematic review. BMJ 2021;375:n2281. 10.1136/bmj.n2281 34670780 PMC8527348

[50] Christodoulou E Ma J Collins GS Steyerberg EW Verbakel JY Van Calster B . A systematic review shows no performance benefit of machine learning over logistic regression for clinical prediction models. J Clin Epidemiol 2019;110:12-22. 10.1016/j.jclinepi.2019.02.004 30763612

[51] Yusuf M Atal I Li J . Reporting quality of studies using machine learning models for medical diagnosis: a systematic review. BMJ Open 2020;10:e034568. 10.1136/bmjopen-2019-034568 32205374 PMC7103817

[52] Wang W Kiik M Peek N . A systematic review of machine learning models for predicting outcomes of stroke with structured data. PLoS One 2020;15:e0234722. 10.1371/journal.pone.0234722 32530947 PMC7292406

[53] Miles J Turner J Jacques R Williams J Mason S . Using machine-learning risk prediction models to triage the acuity of undifferentiated patients entering the emergency care system: a systematic review. Diagn Progn Res 2020;4:16. 10.1186/s41512-020-00084-1 33024830 PMC7531169

[54] Dhiman P Ma J Navarro CA . Reporting of prognostic clinical prediction models based on machine learning methods in oncology needs to be improved. J Clin Epidemiol 2021;138:60-72. 10.1016/j.jclinepi.2021.06.024 34214626 PMC8592577

[55] Dhiman P Ma J Andaur Navarro CL . Risk of bias of prognostic models developed using machine learning: a systematic review in oncology. Diagn Progn Res 2022;6:13. 10.1186/s41512-022-00126-w 35794668 PMC9261114

[56] Araújo ALD Moraes MC Pérez-de-Oliveira ME . Machine learning for the prediction of toxicities from head and neck cancer treatment: A systematic review with meta-analysis. Oral Oncol 2023;140:106386. 10.1016/j.oraloncology.2023.106386 37023561

[57] Sheehy J Rutledge H Acharya UR . Gynecological cancer prognosis using machine learning techniques: A systematic review of the last three decades (1990-2022). Artif Intell Med 2023;139:102536. 10.1016/j.artmed.2023.102536 37100507

[58] Collins GS Whittle R Bullock GS . Open science practices need substantial improvement in prognostic model studies in oncology using machine learning. J Clin Epidemiol 2024;165:111199. 10.1016/j.jclinepi.2023.10.015 37898461

[59] DhimanP MaJ Andaur NavarroCL . Overinterpretation of findings in machine learning prediction model studies in oncology: a systematic review. J Clin Epidemiol 2023;157:120-33. 10.1016/j.jclinepi.2023.03.012 36935090

[60] Andaur Navarro CL Damen JAA Takada T . Systematic review finds “spin” practices and poor reporting standards in studies on machine learning-based prediction models. J Clin Epidemiol 2023;158:99-110. 10.1016/j.jclinepi.2023.03.024 37024020

[61] Chen IY Pierson E Rose S Joshi S Ferryman K Ghassemi M . Ethical Machine Learning in Healthcare. Annu Rev Biomed Data Sci 2021;4:123-44. 10.1146/annurev-biodatasci-092820-114757 34396058 PMC8362902

[62] Ganapathi S Palmer J Alderman JE . Tackling bias in AI health datasets through the STANDING Together initiative. Nat Med 2022;28:2232-3. 10.1038/s41591-022-01987-w 36163296

[63] Kadakia KT Beckman AL Ross JS Krumholz HM . Leveraging Open Science to Accelerate Research. N Engl J Med 2021;384:e61. 10.1056/NEJMp2034518 33761227

[64] Staniszewska S Brett J Simera I . GRIPP2 reporting checklists: tools to improve reporting of patient and public involvement in research. BMJ 2017;358:j3453. 10.1136/bmj.j3453 28768629 PMC5539518

[65] Camaradou JCL Hogg HDJ . Commentary: Patient Perspectives on Artificial Intelligence; What have We Learned and How Should We Move Forward? Adv Ther 2023;40:2563-72. 10.1007/s12325-023-02511-3 37043172 PMC10092909

[66] Finlayson SG Beam AL van Smeden M . Machine Learning and Statistics in Clinical Research Articles-Moving Past the False Dichotomy. JAMA Pediatr 2023;177:448-50. 10.1001/jamapediatrics.2023.0034 36939696

[67] Sounderajah V Ashrafian H Golub RM STARD-AI Steering Committee . Developing a reporting guideline for artificial intelligence-centred diagnostic test accuracy studies: the STARD-AI protocol. BMJ Open 2021;11:e047709. 10.1136/bmjopen-2020-047709 34183345 PMC8240576

[68] Mongan J Moy L Kahn CE Jr . Checklist for Artificial Intelligence in Medical Imaging (CLAIM): A Guide for Authors and Reviewers. Radiol Artif Intell 2020;2:e200029. 10.1148/ryai.2020200029 33937821 PMC8017414

[69] Vasey B Nagendran M Campbell B DECIDE-AI expert group . Reporting guideline for the early-stage clinical evaluation of decision support systems driven by artificial intelligence: DECIDE-AI. Nat Med 2022;28:924-33. 10.1038/s41591-022-01772-9 35585198

[70] Hawksworth C, Elvidge J, Knies S, et al. Protocol for the development of an artificial intelligence extension to the Consolidated Health Economic Evaluation Reporting Standards (CHEERS) 2022. Health Economics; 2023. https://www.medrxiv.org/lookup/doi/10.1101/2023.05.31.23290788

[71] Rivera SC Liu X Chan AW Denniston AK Calvert MJ SPIRIT-AI and CONSORT-AI Working Group . Guidelines for clinical trial protocols for interventions involving artificial intelligence: the SPIRIT-AI Extension. BMJ 2020;370:m3210. 10.1136/bmj.m3210 32907797 PMC7490785

[72] Liu X Rivera SC Moher D Calvert MJ Denniston AK SPIRIT-AI and CONSORT-AI Working Group . Reporting guidelines for clinical trial reports for interventions involving artificial intelligence: the CONSORT-AI Extension. BMJ 2020;370:m3164. 10.1136/bmj.m3164 32909959 PMC7490784

[73] Cacciamani GE Chu TN Sanford DI . PRISMA AI reporting guidelines for systematic reviews and meta-analyses on AI in healthcare. Nat Med 2023;29:14-5. 10.1038/s41591-022-02139-w 36646804

[74] Collins GS Dhiman P Ma J . Evaluation of clinical prediction models (part 1): from development to external validation. BMJ 2024;384:e074819. 10.1136/bmj-2023-074819 38191193 PMC10772854

[75] Riley RD Archer L Snell KIE . Evaluation of clinical prediction models (part 2): how to undertake an external validation study. BMJ 2024;384:e074820. 10.1136/bmj-2023-074820 38224968 PMC10788734

[76] Van Calster B Steyerberg EW Wynants L van Smeden M . There is no such thing as a validated prediction model. BMC Med 2023;21:70. 10.1186/s12916-023-02779-w 36829188 PMC9951847

[77] Moher D Schulz KF Simera I Altman DG . Guidance for developers of health research reporting guidelines. PLoS Med 2010;7:e1000217. 10.1371/journal.pmed.1000217 20169112 PMC2821895

[78] Collins GS Moons KGM . Reporting of artificial intelligence prediction models. Lancet 2019;393:1577-9. 10.1016/S0140-6736(19)30037-6 31007185

[79] Collins GS Dhiman P Andaur Navarro CL . Protocol for development of a reporting guideline (TRIPOD-AI) and risk of bias tool (PROBAST-AI) for diagnostic and prognostic prediction model studies based on artificial intelligence. BMJ Open 2021;11:e048008. 10.1136/bmjopen-2020-048008 34244270 PMC8273461

[80] Gattrell WT Logullo P van Zuuren EJ . ACCORD (ACcurate COnsensus Reporting Document): A reporting guideline for consensus methods in biomedicine developed via a modified Delphi. PLoS Med 2024;21:e1004326. 10.1371/journal.pmed.1004326 38261576 PMC10805282

[81] Olczak J Pavlopoulos J Prijs J . Presenting artificial intelligence, deep learning, and machine learning studies to clinicians and healthcare stakeholders: an introductory reference with a guideline and a Clinical AI Research (CAIR) checklist proposal. Acta Orthop 2021;92:513-25. 10.1080/17453674.2021.1918389 33988081 PMC8519529

[82] Norgeot B Quer G Beaulieu-Jones BK . Minimum information about clinical artificial intelligence modeling: the MI-CLAIM checklist. Nat Med 2020;26:1320-4. 10.1038/s41591-020-1041-y 32908275 PMC7538196

[83] Hernandez-Boussard T Bozkurt S Ioannidis JPA Shah NH . MINIMAR (MINimum Information for Medical AI Reporting): Developing reporting standards for artificial intelligence in health care. J Am Med Inform Assoc 2020;27:2011-5. 10.1093/jamia/ocaa088 32594179 PMC7727333

[84] Scott I Carter S Coiera E . Clinician checklist for assessing suitability of machine learning applications in healthcare. BMJ Health Care Inform 2021;28:e100251. 10.1136/bmjhci-2020-100251 33547086 PMC7871244

[85] Schwendicke F Singh T Lee JH IADR e-oral health network and the ITU WHO focus group AI for Health . Artificial intelligence in dental research: Checklist for authors, reviewers, readers. J Dent 2021;107:103610. 10.1016/j.jdent.2021.103610 33631303

[86] Sendak MP Gao M Brajer N Balu S . Presenting machine learning model information to clinical end users with model facts labels. NPJ Digit Med 2020;3:41. 10.1038/s41746-020-0253-3 32219182 PMC7090057

[87] Stevens LM Mortazavi BJ Deo RC Curtis L Kao DP . Recommendations for Reporting Machine Learning Analyses in Clinical Research. Circ Cardiovasc Qual Outcomes 2020;13:e006556. 10.1161/CIRCOUTCOMES.120.006556 33079589 PMC8320533

[88] Kwong JCC McLoughlin LC Haider M . Standardized Reporting of Machine Learning Applications in Urology: The STREAM-URO Framework. Eur Urol Focus 2021;7:672-82. 10.1016/j.euf.2021.07.004 34362709

[89] de Hond AAH Leeuwenberg AM Hooft L . Guidelines and quality criteria for artificial intelligence-based prediction models in healthcare: a scoping review. NPJ Digit Med 2022;5:2. 10.1038/s41746-021-00549-7 35013569 PMC8748878

[90] Wolff RF Moons KGM Riley RD PROBAST Group† . PROBAST: A Tool to Assess the Risk of Bias and Applicability of Prediction Model Studies. Ann Intern Med 2019;170:51-8. 10.7326/M18-1376 30596875

[91] Moons KGM Wolff RF Riley RD . PROBAST: A Tool to Assess Risk of Bias and Applicability of Prediction Model Studies: Explanation and Elaboration. Ann Intern Med 2019;170:W1-33. 10.7326/M18-1377 30596876

[92] Ibrahim H Liu X Zariffa N Morris AD Denniston AK . Health data poverty: an assailable barrier to equitable digital health care. Lancet Digit Health 2021;3:e260-5. 10.1016/S2589-7500(20)30317-4 33678589

[93] McCradden MD Joshi S Mazwi M Anderson JA . Ethical limitations of algorithmic fairness solutions in health care machine learning. Lancet Digit Health 2020;2:e221-3. 10.1016/S2589-7500(20)30065-0 33328054

[94] Mccradden M, Odusi O, Joshi S, et al. *What’s fair is… fair? Presenting JustEFAB, an ethical framework for operationalizing medical ethics and social justice in the integration of clinical machine learning: JustEFAB.* In: 2023 ACM Conference on Fairness, Accountability, and Transparency. ACM 2023;1505-19. https://dl.acm.org/doi/10.1145/3593013.3594096.

[95] Thibault RT Amaral OB Argolo F Bandrowski AE Davidson AR Drude NI . Open Science 2.0: Towards a truly collaborative research ecosystem. PLoS Biol 2023;21:e3002362. 10.1371/journal.pbio.3002362 37856538 PMC10617723

[96] Riley R Tierney J Stewart L , eds. Individual participant data meta-analysis: a handbook for healthcare research. Wiley, 2021 10.1002/9781119333784.

