# Supplementary material for: TRIPOD+AI statement: updated guidance for reporting clinical prediction models that use regression or machine learning methods
Source: BMJ. 2024 Apr 16;385:e078378. doi: 10.1136/bmj-2023-078378 (PMC11019967; doi:10.1136/bmj-2023-078378)
Supplement: Supplementary file 1 — Supplementary table 1: TRIPOD+AI Expanded Checklist (Explanation and Elaboration Light) [file colg078378.wt1.pdf]

TRIPOD+AI Expanded Checklist (Explanation & Elaboration Light)

Section/Topic	Item		Checklist item
**TITLE**			
Title	1	D;E	**Identify the study as developing or evaluating the performance of a multivariable prediction model, the target population, and the outcome to be predicted**<br>● *Informative titles aid the identification of prediction model studies by potential readers and also systematic reviewers*<br>● *Report an informative title that provides key information about the target population and the outcome being predicted by the model*
**ABSTRACT**			
Abstract	2	D;E	**See TRIPOD+AI for Abstracts checklist**<br>● *Report an abstract addressing each item in the TRIPOD+AI for Abstracts checklist*
**INTRODUCTION**			
Background	3a	D;E	**Explain the healthcare context (including whether diagnostic or prognostic) and rationale for developing or evaluating the prediction model, including references to existing models**<br>● *Describe the healthcare setting where the model is intended to be used or needed*<br>● *Where an existing prediction model is available, provide a clear justification for developing a new model*<br>● *For studies evaluating an existing model, provide the rationale for the evaluation, and provide references to all models being evaluated*
	3b	D;E	**Describe the target population and the intended purpose of the prediction model in the context of the care pathway, including its intended users (e.g., healthcare professionals, patients, public)**<br>● *Describe who is the target population for the developed or evaluated model, e.g., people of a certain age, in a specific country, or with a specific disease*<br>● *Describe the intended purpose of the model, including the clinical decision or guidance the model is intended used to support (e.g., referral for further testing or hospital admission, triage, starting a treatment, or changing a lifestyle) and the point in the care pathway the model is to be intended used*<br>● *Describe who the intended users of the model are, and if the model is for healthcare professionals, patients, public or other*
	3c	D;E	**Describe any known health inequalities between sociodemographic groups**

Section/Topic	Item		Checklist item
			• *In the context of the healthcare setting where the model is intended to be used, describe any known health inequalities between sociodemographic groups in the target population (along with citations to support the health inequalities)*
Objectives	4	D;E	**Specify the study objectives, including whether the study describes the development or validation of a prediction model (or both)**<br>• *Provide an explicit statement of all objectives of the study, describing whether the study is developing a prediction model, evaluating the performance of a prediction model, or both*
**METHODS**			
Data	5a	D;E	**Describe the sources of data separately for the development and evaluation datasets (e.g., randomised trial, cohort, routine care or registry data), the rationale for using these data, and representativeness of the data**<br>• *Provide a description of the source of the data used for model development and evaluation of model performance, including whether the data are (for example) from a randomised trial, a cohort, a registry or from electronic routine healthcare records*<br>• *Specify whether the study is using existing data or is prospectively collecting new data for the purpose of the prediction model study*<br>• *Where existing data are being used (i.e., they were originally collected for a different purpose), provide the rationale for using these data, and comment on the suitability (particularly if data are being used from a different setting or country to the intended target population) and representativeness of these data with respect to the intended target population and context*<br>• *A description of the data sources should be provided for all data sets, and separately for development and evaluation*<br>• *If any synthetic data have been used, then provide reasons as to why, and provide all details on how the synthetic data have been created (and code, see item 18f) and used in the study*
	5b	D;E	**Specify the dates of the collected participant data, including start and end of participant accrual; and, if applicable, end of follow-up**<br>• *Specify the start and end dates of the period for which the participants or the used data were selected*<br>• *For models predicting prognosis, the duration of follow-up is important so report the date of end of follow-up*
Participants	6a	D;E	**Specify key elements of the study setting (e.g., primary care, secondary care, general population) including the number and location of centres**<br>• *Describe the healthcare setting, and where the participants in the study were recruited from*<br>• *Report the geographical location (at a minimum, the country) and centres (including the number of centres) of the study*
	6b	D;E	**Describe the eligibility criteria for study participants**<br>• *The eligibility criteria for participants should be reported to understand the potential applicability and generalisability of the prediction model*

Section/Topic	Item		Checklist item
			• *This includes reporting any restrictions of continuous variables, e.g., age range used to define the eligibility of the included participants*
	6c	D;E	**Give details of any treatments received, and how they were handled during model development or evaluation, if relevant**<br>• *Any treatments received before or at the start of follow-up should be reported, and whether and how this was handled during the development or evaluation of the prediction model (if relevant)*<br>• *Any treatments received between the moment the prediction model is used and the measurement of the outcome, that could modify the probability of the outcome, should be reported (if relevant)*
Data preparation	7	D;E	**Describe any data pre-processing and quality checking, including whether this was similar across relevant sociodemographic groups**<br>• *Describe any data cleaning steps, this includes any feature engineering, transformation of raw data, feature reduction and data quality checks. All code used for data cleaning should be made available (see item 18f)*<br>• *For analyses using data from multiple sources (e.g., data from different studies, cohorts, or registries), describe any harmonisation (e.g., of outcome and predictors)*<br>• *Confirm whether all data pre-processing/data cleaning steps were similar across key socio demographic groups, if relevant*<br>• *If the data pre-processing/data cleaning steps are extensive, consider reporting this information in the supplementary material*
Outcome	8a	D;E	**Clearly define the outcome that is being predicted and the time horizon, including how and when assessed, the rationale for choosing this outcome, and whether the method of outcome assessment is consistent across sociodemographic groups**<br>• *For diagnostic prediction models, the outcome should be clearly defined, including whether a (widely accepted) reference standard (ground truth) was used to determine the presence or absence of the outcome*<br>• *For prognostic models, i.e., models predicting an outcome in the future, authors should report the time-horizon of the outcome prediction. For example, predicting the 28-day risk of mortality following cardiothoracic surgery, or the 10-year risk of fractures in patients with osteoporosis. Also, the frequency of outcome assessment during follow-up should be reported*<br>• *If standard definitions are used, e.g., using ICD[1] codes, this should be stated and referenced*<br>• *Any discrepancies in the outcome assessment across socio-demographic groups should be reported*<br>• *In some instances, it may be necessary to confirm that no predictors were used to define the outcome or are a proxy for the outcome*
	8b	D;E	**If outcome assessment requires subjective interpretation, describe the qualifications and demographic characteristics of the outcome assessors**<br>• *For outcomes that require a subjective interpretation (e.g., interpreting the results from an imaging test, describe the number, qualification, and demographic characteristics of the outcome assessors)*

[1] ICD stands for International Classification of Diseases

Section/Topic	Item		Checklist item
			• *If the measurement and interpretation of the outcome require (additional) training or specific instructions, these should be reported.*<br>• *If extensive, consider reporting this information in the supplementary material*
	8c	D;E	**Report any actions to blind assessment of the outcome to be predicted**<br>• *The outcome being predicted should be assessed blind to information about the predictors – particularly relevant for outcomes requiring a subjective interpretation thereby avoiding data (label) leakage*<br>• *If appropriate, authors should describe which information was available to the outcome assessors and report any specific actions to blinding the outcome assessment*
Predictors	9a	D	**Describe the choice of initial predictors (e.g., literature, previous models, all available predictors) and any pre-selection of predictors before model building**<br>• *Provide details on how the initial list of predictors were considered for inclusion in the model building, and whether they were chosen based on a (systematic) review of the literature, clinical input (domain experts), or simply whether using all predictors in the available data*<br>• *If any pre-selection of predictors, before model building, was carried out, then provide details how this was done. For example, were predictors omitted for model building due to high amounts of missing data, or predictors not considered plausibly (clinically) related to the outcome being predicted*<br>• *The list of initial predictors may be extensive, in these instances reporting these in the supplementary material is advisable*
	9b	D;E	**Clearly define all predictors, including how and when they were measured (and any actions to blind assessment of predictors for the outcome and other predictors)**<br>• *All predictors included in the modelling should be clearly defined, along with units of measurement, and all categories for categorical predictors, so that readers and others can replicate, implement, or evaluate the performance of the model*<br>• *Details on how and when the predictor values were measured. Note that predictors should be measured before or at the time the model is intended to be used*<br>• *For predictors requiring subjective interpretation, it may be important to interpret this blind to the values of other predictors considered in the modelling (e.g., avoiding data leakage). Authors should report any actions to blind the assessment of the predictor measurement to other predictors*<br>• *Specifically for diagnostic models, the measurement of the predictors should be done without knowledge of the outcome of the individual as this could artificially inflate the association between the predictors and the outcome. Authors should report any actions to blind the assessment of the predictor measurements to the outcome value*<br>• *In some instances, the number of predictors can be very large and thus reporting them all in the main manuscript is unhelpful, in these instances, it is still important to clearly define all the predictors, and reporting this in the supplementary material should be considered*

Section/Topic	Item		Checklist item
	9c	D;E	**If predictor measurement requires subjective interpretation, describe the qualifications and demographic characteristics of the predictor assessors**<br>• *For predictors that require a subjective interpretation (e.g., interpreting the results from an imaging test), the qualifications and demographic characteristics of the predictor assessors should be reported*<br>• *If the measurement and interpretation require (additional) training or specific instructions, then these should be reported. This could be reported in the supplementary material*
Sample size	10	D;E	**Explain how the study size was arrived at (separately for development and evaluation), and justify that the study size was sufficient to answer the research question. Include details of any sample size calculation**<br>• *Describe how the sample size was determined – this should be done separately for determining the sample size needed for model development and the sample size needed to evaluate the performance of the model irrespective of whether data are being prospectively collected or using existing data*<br>• *Provide details and all estimates used in any sample size calculation*<br>• *If no formal sample size calculation was done, e.g., all available data were used, provide a justification whether the size of the data was sufficient to answer the research question*
Missing data	11	D;E	**Describe how missing data were handled. Provide reasons for omitting any data**<br>• *Missing data is an omnipresent problem. Authors should report for each predictor being considered for inclusion in the model the number of missing values*<br>• *The handling of missing values should be reported, including any assumptions for the reason of the missingness*<br>• *If individuals (or predictors) have been omitted due to the missing values, this should be reported, and reasons given*<br>• *If missing values have been imputed, then full details of the method for imputing any missing values should be reported*<br>• *If missing values have been imputed confirm it was done separately for the training and any test data (i.e., avoiding leakage)*
Analytical methods	12a	D	**Describe how the data were used (e.g., for development and evaluation of model performance) in the analysis, including whether the data were partitioned, considering any sample size requirements**<br>• *Describe how the available data were used to develop the model and to evaluate model performance, including whether and how the data were partitioned, and the reasons for partitioning the data (e.g., model development, hyperparameter tuning, evaluating model performance, internal-external cross-validation)*<br>• *If the data has been partitioned, report whether sample size requirements (see item 10) were considered during the partitioning, and whether the size of the partitioned data are sufficient to carry out the analyses and answer the research question*<br>• *If the data has been partitioned into training (including any hyperparameter tuning data) and test data, confirm that there has been no data leakage*

Section/Topic	Item		Checklist item
			• *If the data contain multiple records or samples from the same individual, and if the data has been partitioned into training (including any hyperparameter tuning data) and test data, confirm there has been no leakage of individuals across any of the partitioned data or if not, how describe how this was handled in the analysis (see item 12c)*
	12b	D	**Depending on the type of model, describe how predictors were handled in the analyses (functional form, rescaling, transformation, or any standardisation)**<br>• *For any predictors that have been transformed during the analysis, i.e., rescaled, or standardised, describe how this was done*<br>• *For any categorical predictors, where collapsing of categories has been carried out, e.g., due to small sample size/too few outcome events, provide the details and reasons*
	12c	D	**Specify the type of model, rationale[2], all model-building steps, including any hyperparameter tuning, and method for internal validation**<br>• *Clearly specify the type of model (or models) being developed (e.g., logistic regression, Cox regression, random forest, neural network) and provide a rationale for using each model building method – consider the type of outcome being predicted and how the prediction model will be implemented in practice*<br>• *For each model, clearly describe all the steps in the model building, including any hyperparameter tuning, what hyperparameters have been tuned and how this was done. If many model building approaches are being applied and word limits prohibit a full description, then use supplementary material to provide the details*<br>• *For studies that are developing more than one model (e.g., using different model building methods), clearly describe the criteria to choose which is the model being put forward (if any), see item 12e and item 23 on model performance)*<br>• *The internal validation approach (to evaluate model performance) during model development should be clearly described, e.g., was k-fold cross validation or bootstrapping used. Clarify whether all model building steps (including hyperparameter tuning) was replayed during the method of internal evaluation*<br>• *Clearly describe any methods (e.g., bootstrapping) used to examine model stability (e.g., in terms of predictor selection, predictive performance and individual predictions) (Riley & Collins, Biom J 2023; 65: 2200302 [DOI: 10.1002/bimj.202200302])*<br>• *If the data contain multiple records or samples from the same individual, describe how this was handled in the model building and internal validation (e.g., if k-fold cross-validation was used, confirm if all records/samples for an individual were included in the same fold (e.g., avoiding data leakage)*
	12d	D;E	**Describe if and how any heterogeneity in estimates of model parameter values and model performance was handled and quantified across clusters (e.g., hospitals, countries). See TRIPOD-Cluster for additional considerations[3]**

[2] Separately for all model building approaches.
[3] TRIPOD-Cluster is a checklist of reporting recommendations for studies developing or validating models that explicitly account for clustering or explore heterogeneity in model performance (e.g., at different hospitals/centres).

Section/Topic	Item		Checklist item
			• *If the analysis has accounted for any clustering in the data (e.g., from combining individual participant data from multiple studies, or data clustered by medical centre/hospital, or country) during the model development or evaluation of model performance, the rationale and methods used to account for clustering should be clearly described*<br>• *For specific reporting recommendations for prediction model studies that have accounted for clustering and heterogeneity in model parameter values and performance, authors should consult the TRIPOD-Cluster checklist (Debray et al, BMJ 2023; 380: e071018 [DOI: 10.1136/bmj-2022-071018])*
	12e	D;E	**Specify all measures and plots used (and their rationale) to evaluate model performance (e.g., discrimination, calibration, clinical utility) and, if relevant, to compare multiple models**<br>• *Report all the measures used to evaluate model performance. It is generally expected that as a minimum, model discrimination and calibration (including calibration plots) are presented*<br>• *If the prediction model is predicting a time-to-event outcome, then clearly describe the measures and methods that have been used to account for the time-to-event nature (i.e., censoring). Similarly, the handling of any competing risks should also be stated (if applicable)*<br>• *For prognostic models, report all time-points at which the model's predictive performance was evaluated*<br>• *Report the methods used for graphical displays of model performance, such as calibration plots (with smooth calibration curves) and decision curves*<br>• *If multiple models are being compared, i.e., comparing against an existing model or comparing multiple modelling approaches, then the methods used for comparing these models, and the criteria for making any judgements on superior performance should be clearly explained*
	12f	E	**Describe any model updating (e.g., recalibration) arising from the model evaluation, either overall or for particular sociodemographic groups or settings**<br>• *If the model is updated following the validation, such as recalibration or refitting – whether in the entire cohort or in a specific socio demographic group, then provide details on the methods used to update the model*
	12g	E	**For model evaluation, describe how the model predictions were calculated (e.g., formula, code, object, application programming interface)**<br>• *For studies evaluating an existing model in a separate data set (i.e., an external validation study), provide details on how the individual predictions from the model were calculated. If a model is not freely/publicly available, explain how the predictions were obtained*<br>• *If a regression model equation was being evaluated, provide details of this equation (e.g., consider presenting this equation, provide a citation to the original study that developed the equation)*

Section/Topic	Item		Checklist item
			● *For studies evaluating a prediction model where there is no equation (e.g., a neural network, random forest), provide details on how the predictions were made, e.g., code, software object, API, and where can this be found (i.e., URL[4], DOI[5])*<br>● *If individual predictions from the model were used to create risk groups or classifications (that were not specified in the model development) then details on how and why this was done should be reported (see item 15)*
Class imbalance	13	D;E	**If class imbalance methods were used, state why and how this was done, and any subsequent methods to recalibrate the model or the model predictions**<br>● *If class imbalance methods (e.g., under/over sampling, SMOTE[6]) have been used, then provide a rationale for doing so, and how this was done – considering any impact on sample size (e.g., for undersampling methods)*<br>● *Imbalance corrections have an impact on model calibration (van den Goorbergh et al, J Am Med Inform Assoc 2022; 29: 1525--1534 [DOI: 10.1093/jamia/ocac093]), yielding probability estimates that are too high (which also has an impact on defining any risk groups), describe the methods used to recalibrate the model or the model predictions*
Fairness	14	D;E	**Describe any approaches that were used to address model fairness and their rationale**<br>● *Fairness refers to ensuring that a prediction model does not discriminate against individuals or groups, for example based on personal attributes such as race, gender, age and all approaches used to address fairness should be clearly explained along with their rationale*<br>● *It is important to ensure the data contains representative groups (of the target population) when developing the model and evaluating its performance and researchers should attempt to demonstrate this*<br>● *If the prediction model is developed using data with underrepresented groups or particular groups not included, then evaluation in these groups in representative data is needed to evaluate the model in these groups, as to increase generalisability to more groups of individuals beyond those in the development and evaluation data*
Model output	15	D	**Specify the output of the prediction model (e.g., probabilities, classification). Provide details and rationale for any classification and how the thresholds were identified**<br>● *Most models output a probability estimate for an individual, whilst some models turn the output into a classification (e.g., into low- or high-risk groups), this should be clearly stated. If classification or risk groups have been created, then the rationale for doing so in the context of the care pathway and how these risk groups inform any clinical decisions should be made*<br>● *For models producing a classification or risk groups, this should be clearly reported, and any thresholds (e.g., range of estimated probabilities defining the groups) should be specified (whether these are based on the literature, clinical guidelines, statistical considerations or ad-hoc)*

[4] URL stands for uniform resource locator
[5] DOI stands for digital object identifier
[6] SMOTE stands for synthetic minority oversampling technique

Section/Topic	Item		Checklist item
			• *If uncertainty intervals for individual prediction model outputs have been presented then provide details on how this was done (e.g., using the variance-covariance matrix of parameter estimates or conformal prediction)*
Development versus evaluation	16	D;E	**Identify any differences between the development and evaluation data in healthcare setting, eligibility criteria, outcome, and predictors**<br>• *Prediction models developed in one setting, centre or country are not necessarily useful in a different setting, centre, or country. Eligibility criteria, outcome and predictors definitions might (intentionally) differ between data from different sources. Describing any differences between the development data and data used to evaluate model performance is useful to understand and interpret the performance and generalisability of the model in the context of the original model development data*
Ethical approval	17	D;E	**Name the institutional research board or ethics committee that approved the study and describe the participant-informed consent or the ethics committee waiver of informed consent**<br>• *If the study has no institutional research board or ethics approval, then clearly state so, with reasons*
**OPEN SCIENCE**			
Funding	18a	D;E	**Give the source of funding and the role of the funders for the present study**<br>• *Provide details on whether the study was funded and provide any details on the role the funder had in the study.*<br>• *Provide any additional funding sources all authors*
Conflicts of interest	18b	D;E	**Declare any conflicts of interest and financial disclosures for all authors**<br>• *Disclose any of the authors' relationships or activities that readers could consider pertinent or that may have influenced the study design, conduct, interpretation, or reporting*
Protocol	18c	D;E	**Indicate where the study protocol can be accessed or state that a protocol was not prepared**<br>• *Provide all details on the availability of the study protocol, including where the study protocol can be found (e.g., publication details, in supplementary material, publicly available in a repository such as on the Open Science Framework), including a URL or DOI*<br>• *Clearly state if no study protocol was developed or publicly available (and reasons)*<br>• *If there are any notable deviations from what was specified in the study protocol, provide a summary and reasons for the deviation*
Registration	18d	D;E	**Provide registration information for the study, including register name and registration number, or state that the study was not registered**<br>• *If the study has been registered (e.g., on clinicaltrials.gov, Open Science Framework), then provide details on the registration number, the name of the register and a link to the registration (including any DOI)*<br>• *Clearly state if the study has not been registered*

Section/Topic	Item		Checklist item
Data sharing	18e	D;E	**Provide details of the availability of the study data**<br>● *Provide details on the availability of the study data, including where the data can be found (e.g., public repository, URL, DOI), how it can be retrieved, any conditions or restrictions on obtaining and using the data. A data dictionary should accompany any shared data.*<br>● *If data cannot be shared, provide reasons as to why*<br>● *Avoid platitudes such as 'Data available upon reasonable request' without specifying conditions for what constitutes a reasonable request*
Code sharing	18f	D;E	**Provide details of the availability of the analytical code**[7]<br>● *Provide all details on the availability of the analytical code (and documentation on how to run the code), including where the code can be found (e.g., code repository, DOI, link), how it can be retrieved, any conditions or licences to obtain and use the code should be reported (and version)*<br>● *The analytical code is all code needed to replicate (in principle) all the reported results and findings of the study (including any code for data cleaning). The software and any packages needed to reproduce (in principle) the study findings should be reported (including any version numbers). In some instances, more details on the computing environment may need to be reported (e.g., hardware, operating system, CPU, RAM)*
**PATIENT & PUBLIC INVOLVEMENT**			
Patient & Public Involvement	19	D;E	**Provide details of any patient and public involvement during the design, conduct, reporting, interpretation, or dissemination of the study or state no involvement**<br>● *Describe how patients or public were involved in the planning, design, conduct, reporting or dissemination of the study and its findings.*<br>● *Were the findings of the study presented to patients or the public?*<br>● *Consider using the GRIPP2 statement to report patient and public involvement in the research (Staniszewska et al, BMJ 2017; j3453 [DOI: 10.1136/bmj.j3453])*<br>● *If no patients or public were involved in any aspect of the study, then clearly state so*
**RESULTS**			
Participants	20a	D;E	**Describe the flow of participants through the study, including the number of participants with and without the outcome and, if applicable, a summary of the follow-up time. A diagram may be helpful**

[7] This relates to the analysis code, e.g., any data cleaning, feature engineering, model building, evaluation.

Section/Topic	Item		Checklist item
			• *A flow diagram can be useful to describe the flow of participants through a study, where the entry point to the flow diagram is the source of participants, and then successive steps can relate to eligibility criteria, follow-up (if applicable). and data availability*<br>• *Other useful information to present in the flow diagram include the number of participants with missing values, and the number of outcome events*<br>• *For studies of prognosis or diagnosis with delayed reference testing, a summary of the follow-up time should be reported (e.g., median follow-up, and range)*
	20b	D;E	**Report the characteristics overall and, where applicable, for each data source or setting, including the key dates, key predictors (including demographics), treatments received, sample size, number of outcome events, follow-up time, and amount of missing data. A table may be helpful. Report any differences across key demographic groups**<br>• *Report, possibly using a table, a summary of all data sets used, including the distribution of outcomes, predictors (e.g., mean/median, standard deviation/interquartile range, frequency), any treatments received, the sample size (and number of outcome events, summary of the follow-up time, and for each predictor, the number and proportion of missing values*<br>• *If relevant, it may be useful to report any differences across key demographic groups of interest*
	20c	E	**For model evaluation, show a comparison with the development data of the distribution of important predictors (demographics, predictors, and outcome).**<br>• *For studies evaluating the performance of an existing model (including those within a model development study) provide a comparison of the distribution of important variables (e.g., mean/median, standard deviation/interquartile range, frequency), such as demographics, predictors in the model, and outcome, including proportion of missing values. This is probably best presented in a table and consider reporting this by outcome status*
Model development	21	D;E	**Specify the number of participants and outcome events in each analysis (e.g., for model development, hyperparameter tuning, model evaluation)**<br>• *The sample size (including the number of outcome events) should be reported for each analysis (i.e., each model development, each model evaluation), as they can often vary across different analyses in a prediction model study (e.g., after data partitioning, model hyperparameter tuning), and particularly in the presence of missing data*<br>• *If the data contain multiple samples or records for an individual report also report the number of individuals*
Model specification	22	D	**Provide details of the full prediction model (e.g., formula, code, object, API) to allow predictions in new individuals and to enable third-party evaluation and implementation, including any restrictions to access or re-use (e.g., freely available, proprietary)**[8]<br>• *The 'product' of a prediction model development study is the prediction model. It is therefore important to provide details on the model, and how it can be used to allow predictions for new individuals to be made. For example, provide the equation for a*

[8] This relates to the code to implement the model to get estimates of risk for a new individual.

Section/Topic	Item		Checklist item
			regression model, for models developed using methods where the model cannot be 'written down' as an equation, provide details on the availability of code, software objects or API so that others can evaluate this model in their own data, or implement it in daily practice<br>● *If multiple models have been developed, then provide details on the availability of all models*<br>● *Explain how to use the model to allow others to make predictions in new individuals.*<br>● *Provide details of any hardware requirements, and software (and packages) to enable third-party testing, implementation and monitoring*<br>● *If a model cannot be made publicly available (e.g., for commercial reasons), this should be clearly reported, and any conditions on gaining access to the model to enable predictions to be calculated for new individuals and third-party evaluation should be reported*
Model performance	23a	D;E	**Report model performance estimates with confidence intervals, including for any key subgroups (e.g., sociodemographic). Consider plots to aid presentation**<br>● *Estimates of all model performance measures described in item 12e should be presented along with confidence intervals.*<br>● *Report model performance estimates for the overall population and for any key groups (e.g., sex, ethnicity) of interest (e.g., as part of fairness checks) with confidence intervals*<br>● *Use plots to present and aid evaluation, such as calibration plots (with smooth calibration curves and distributions of predicted values) and decision curves*<br>● *Report performance estimates for all evaluations undertaken (e.g., in development data; in evaluation data; from internal validation process, etc), including at each time-point examined (for prognostic models)*<br>● *Report any examinations of model stability, e.g., in terms of performance estimates and variability of individual predictions across models developed in bootstrap samples (Riley & Collins, Biom J 2023; 65: 2200302 [DOI: 10.1002/bimj.202200302])*<br>● *Clearly indicate which data have been used to present each performance estimate*
	23b	D;E	**If examined, report results of any heterogeneity in model performance across clusters. See TRIPOD Cluster for additional details**<br>● *If the evaluation of model performance has accounted for any clustering in the data (e.g., from combining individual participant data from multiple studies, or data clustered by centre/hospital, or country), the results should be reported, along with confidence intervals (see item 23a)*<br>● *For specific reporting recommendations for prediction model studies that have accounted for clustering and heterogeneity in model performance, authors should consult the TRIPOD-Cluster checklist (Debray et al, BMJ 2023; 380: e071018 [DOI: 10.1136/bmj-2022-071018])*
Model updating	24	E	**Report the results from any model updating, including the updated model and subsequent performance**<br>● *If the prediction model has been updated (e.g., recalibrated, re-fit) following the validation, details of the updated prediction model to enable third-party evaluation and implementation, including any restrictions to access or re-use should be reported (see item 22)*

Section/Topic	Item		Checklist item
			● *The performance of the updated model should be reported (see items 23a, potentially 23b)*
**DISCUSSION**			
Interpretation	25	D;E	**Give an overall interpretation of the main results, including issues of fairness in the context of the objectives and previous studies**<br>● *Interpretation of the study results places the findings in context of other evidence. If there are existing models, then discuss the findings in the context of these existing studies*<br>● *For studies evaluating the performance of an existing prediction model, if existing studies have evaluated the performance of the model, then it's important to discuss and summarise these findings and place them in context*<br>● *Ensure the interpretation of the findings do not go beyond the findings reported from the development and evaluation of the model to prevent overinterpretation or 'spin'*<br>● *It is useful for the reader to understand how performance of the model in the evaluation data compares to the performance of the model in any other evaluation studies of that model. When the results diverge, possible reasons for the difference in model performance should be discussed*
Limitations	26	D;E	**Discuss any limitations of the study (such as a non-representative sample, sample size, overfitting, missing data) and their effects on any biases, statistical uncertainty, and generalizability**<br>● *Acknowledgement of limitations is an important aspect of any scientific paper – and can refer to any aspect of the study design, conduct or analysis. Provide a meaningful discussion of the study limitations factoring in any concerns related to representativeness of the data used in the analysis, sample size, overfitting and missing data/data quality*
Usability of the model in the context of current care	27a	D	**Describe how poor quality or unavailable input data (e.g., predictor values) should be assessed and handled when implementing the prediction model**<br>● *Authors should comment on how to handle unavailable predictor values at the moment the model is intended to be used as part of the care pathway in daily practice. Any strategies to impute missing values at the moment the model is intended to be used should also be evaluated (and thus mentioned in the Methods and Results)*<br>● *Similarly, at the point of implementation, authors should discuss (if relevant) the handling of poor quality input data (e.g., image resolution, data format)*
	27b	D	**Discuss whether users will be required to interact in the handling of the input data or use of the model, and what level of expertise is required of users**<br>● *Provide details on how users are expected or required to interact with the prediction model for the model to be used as intended, for example any considerations for handling the input data*<br>● *Is any expertise or training needed or required to use the model, handle or collect the input data, and if so, provide details*

Section/Topic	Item		Checklist item
	27c	D;E	**Discuss any next steps for future research, with a specific view to applicability and generalizability of the model**<br>● *Are further evaluations of the model needed, e.g., in different populations or subgroups, or is the model ready for evaluation in clinical trials, or implementation as part of the care pathway*

From: Collins GS, Moons KGM, Dhiman P, et al. *BMJ* 2024;385:e078378. doi:10.1136/bmj-2023-078378
