# Supplementary material for: TRIPOD+AI statement: updated guidance for reporting clinical prediction models that use regression or machine learning methods
Source: BMJ. 2024 Apr 16;385:e078378. doi: 10.1136/bmj-2023-078378 (PMC11019967; doi:10.1136/bmj-2023-078378)
Supplement: Supplementary file 3 — Supplementary table 3: Aggregated TRIPOD+AI responses from Delphi round 1 [file colg078378.wt3.pdf]

Supplementary Table 3: Aggregated TRIPOD+AI responses from Delphi round 1

			Can be omitted	Possibly include	Desirable for inclusion	Essential for inclusion	Desirable + Essential
1. Title		Identify the study as developing and/or validating (i.e., testing) a multivariable prediction model, the target population, and the outcome to be predicted.	1%	5%	11%	83%	94%
2. Abstract		Provide a summary of objectives, study design or data sources, setting, participants, sample size, predictors/features, outcome, analytical methods, intended use of the prediction model, results, and conclusions.	1%	1%	11%	86%	97%
3. Introduction	a. Background	i. Explain the medical context (including whether diagnostic or prognostic) and rationale for developing or validating the prediction model, including references to existing (AI or non-AI) models, and the main advantages of the used design and analyses.	1%	4%	23%	72%	95%
		ii. Explain the intended purpose (e.g., for prognosis or diagnostic predictions) and use for the AI model in the context of the clinical pathway, including its intended users (e.g., healthcare professionals, patients, public).	2%	4%	25%	69%	94%
	b. Objectives	Specify the study objectives, including whether the study describes the development or validation (e.g., testing) of the model or both.	0%	1%	8%	91%	99%
4. Methods	a. Sources of data	i. Describe the study design or source of data (e.g., randomized trial, cohort, routine care or registry data), separately for the development and validation (test) datasets, if applicable.	0%	2%	8%	90%	98%
		ii. Describe the origin of the data, and how data were identified, requested and collected.	0%	8%	20%	72%	92%
		iii. Specify the key dates of the collected participant data, including start and of participant/data accrual; and, if applicable, end of follow-up.	2%	9%	31%	57%	88%

Supplementary Table 3: Aggregated TRIPOD+AI responses from Delphi round 1

			Can be omitted	Possibly include	Desirable for inclusion	Essential for inclusion	Desirable + Essential
	b. Participants	i. Specify key elements of the study setting (e.g., primary care, secondary care, general population) including the number and location of centres or data sources.	0%	6%	28%	66%	94%
		ii. Describe the eligibility criteria for participants or data sources: how, where, and when potentially eligible participants were identified (e.g., symptoms, results from previous tests, inclusion in the registry, patient-care setting, location).	1%	4%	16%	79%	95%
		iii. Give details of treatments received, and how they were handled, if relevant.	5%	10%	41%	45%	86%
	c. Data preparation (predictors/feature and outcome/label)	Describe any data pre-processing steps, including any cleaning, harmonisation, sampling, linkage, de-identification methods, and quality checks.	2%	6%	21%	72%	93%
	d. Outcome/labelling	i. Clearly define the outcome (e.g., ground truth or reference standard) that is predicted by the prediction model (including the time horizon), including how and when assessed and the rationale for choosing this outcome measurement (if alternatives exist).	0%	0%	4%	96%	100%
		ii. Describe the qualifications of the assessors of the outcomes which require subjective interpretation and whether additional materials were used.	2%	13%	41%	44%	85%
		iii. Report any actions to blind assessment of the outcome to be predicted (e.g., details on what, if any, information was used to assess/label the outcome).	3%	14%	35%	48%	83%
		iv. Describe any measurement of inter- and intra- rater variability for outcomes requiring subjective interpretation (e.g., imaging), including methods to mitigate variability or resolve discrepancies.	2%	15%	42%	41%	83%

Supplementary Table 3: Aggregated TRIPOD+AI responses from Delphi round 1

			Can be omitted	Possibly include	Desirable for inclusion	Essential for inclusion	Desirable + Essential
	e. Predictors	i. Clearly define all predictors/features used in developing the multivariable prediction model, including how and when they were measured. Consider using supplementary material for large numbers of predictors.	0%	5%	12%	83%	95%
		ii. Report the source of predictors and any known biases that may be associated with them.	4%	13%	34%	49%	83%
		iii. Describe the qualifications of the assessors of the predictors/features which require subjective interpretation and whether additional materials were used.	7%	25%	36%	32%	68%
		iv. Report any actions to blind assessment of predictors for the outcome and other predictors.	7%	21%	34%	39%	73%
		v. Describe any measurement of inter- and intra- rater variability for predictors requiring subjective interpretation (e.g., imaging), including methods to mitigate variability and/or resolve discrepancies.	5%	24%	36%	34%	70%
	f. Sample size	Explain how the study size was arrived at (and provide a justification that the study size was sufficient). Report assumptions and estimates to support any sample size calculation.	3%	11%	28%	58%	86%
	g. Missing data	Describe how missing data were handled (e.g., complete-case analysis, single imputation, multiple imputation) with details of any imputation or other data augmentation method.	0%	2%	16%	81%	97%
	h. Analytical methods	i. Consider a diagram to illustrate the analytical processes.	10%	19%	38%	33%	71%
		ii. Describe how predictors/features were handled in the analyses (functional form and any standardisation).	1%	7%	25%	67%	92%
		iii. Describe any pre-selection of predictors/features prior to model building.	1%	6%	23%	70%	93%

Supplementary Table 3: Aggregated TRIPOD+AI responses from Delphi round 1

			Can be omitted	Possibly include	Desirable for inclusion	Essential for inclusion	Desirable + Essential
		iv. Describe any predictor/feature rescaling or transformations prior to model building.	3%	10%	29%	58%	87%
		v. Specify the type of model, all model-building procedures (including any predictor selection), and method for internal validation (e.g., bootstrapping, cross-validation).	0%	4%	9%	88%	97%
		vi. Describe any ensemble techniques (e.g., to combine model predictions), if applicable.	4%	11%	24%	62%	86%
		vii. Provide a detailed description of the model, including inputs, outputs, all intermediate layers and connections.	4%	12%	25%	59%	84%
		viii. Describe the initialization of model parameters (e.g., randomization, transfer learning).	5%	10%	30%	54%	84%
		ix. Details of model training approaches, including hyperparameters, number of models trained, used data sets.	2%	6%	19%	73%	92%
		x. Describe how any heterogeneity in the model parameter values was handled across clusters (e.g., hospitals, countries).	2%	13%	38%	47%	85%
		xi. Describe how any heterogeneity in model performance was handled and quantified across clusters (e.g., hospitals, countries).	3%	12%	38%	47%	85%
		xii. Specify all measures used to assess model performance (e.g., discrimination, calibration) and, if relevant, to compare multiple models.	1%	2%	9%	88%	97%
		xiii. Describe any model updating (e.g., recalibration) arising from the validation (testing), either overall or for particular populations or settings.	2%	4%	25%	68%	93%
		xiv. Describe the method of selecting the final model.	1%	3%	13%	84%	97%
		xv. Describe methods for explainability or interpretability and how they were validated.	4%	13%	33%	49%	82%

Supplementary Table 3: Aggregated TRIPOD+AI responses from Delphi round 1

			Can be omitted	Possibly include	Desirable for inclusion	Essential for inclusion	Desirable + Essential
		xvi. For external validation/testing (outside the model development study), describe how the predictions by the model were calculated (e.g., using code made publicly available on GitHub).	4%	8%	24%	64%	88%
	i. Risk groups	Provide details on how risk groups were created, if done.	5%	15%	29%	51%	80%
	j. Model development (e.g., training) versus validation (e.g., testing)	For external validation (testing), identify any differences from the development (training) data in setting, eligibility, criteria, outcome, and predictors.	1%	4%	15%	81%	96%
	k. Software	Provide details of any software libraries, frameworks, or packages used.	2%	6%	19%	73%	92%
5. Results	a. Participants	i. Describe the flow of participants through the study, including the number of participants with and without the outcome and, if applicable, a summary of the follow-up time. A diagram may be helpful.	1%	5%	19%	75%	94%
		ii. Report the characteristics overall and where applicable for each data source or setting, including the key dates, key predictors/features (including demographics, ethnicity), treatments received, sample size, number of outcome events, follow-up time, and amount of missing data.	1%	4%	20%	76%	96%
		iii. For external validation (testing), show a comparison with the development data of the distribution of important predictors/features (demographics, predictors, and outcome).	2%	9%	22%	67%	89%
	b. Model development	Specify the number of participants and outcome events in each analysis (e.g., model building, parameter tuning, testing).	1%	2%	23%	74%	97%

Supplementary Table 3: Aggregated TRIPOD+AI responses from Delphi round 1

			Can be omitted	Possibly include	Desirable for inclusion	Essential for inclusion	Desirable + Essential
	c. Model specification	i. Provide details on the full prediction model to allow predictions for individuals to allow third-party evaluation and implementation (e.g., regression coefficients, input parameters, sharing of code/any dependencies). Provide reasons for not sharing code.	2%	7%	23%	67%	90%
		ii. Specify the output of the AI model (e.g., probabilities, classification, risk grouping).	3%	3%	9%	85%	94%
	d. Model performance	i. Report performance measures (with confidence intervals, CIs) for the prediction model.	0%	2%	8%	90%	98%
		ii. Report results of any heterogeneity across clusters in model performance.	2%	13%	38%	46%	84%
		iii. Describe the results of any analysis of performance errors and how errors were identified, where applicable. If no such analysis was planned or done, explain why not.	4%	21%	37%	38%	75%
	e. Model updating	Report the results from any model updating (including the updated model and subsequent performance), overall and for each cluster (e.g., hospital, if done).	6%	11%	28%	55%	83%
	f. Usability of the model	i. Explain how (and when in the clinical pathway) to use the prediction AI model to permit third-party testing and implementation.	7%	14%	24%	55%	79%
		ii. Describe how the AI model will be integrated into the target setting and clinical pathway, including any onsite or offsite requirements (e.g., hardware/software requirements).	12%	20%	34%	34%	68%
		iii. Describe how poor quality or unavailable input data will be assessed and handled when implementing the AI model.	8%	18%	38%	36%	74%
		iv. Specify whether human-AI interaction will be required in the handling of the input data (e.g., steps required to go from the raw clinical data into a form usable by the model), and what level of expertise is required of users.	11%	18%	30%	41%	71%

Supplementary Table 3: Aggregated TRIPOD+AI responses from Delphi round 1

			Can be omitted	Possibly include	Desirable for inclusion	Essential for inclusion	Desirable + Essential
	g. Sensitivity analysis	Report results from any subgroup or sensitivity analysis.	2%	16%	35%	48%	83%
6. Discussion	a. Limitations	Discuss any limitations of the study (such as a non-representative sample, sample size, overfitting, missing data) and their effects on biases, statistical uncertainty, and generalizability.	0%	2%	9%	90%	99%
	b. Interpretation	i. For external validation (testing), discuss the results with reference to performance in the development data, and any other validation data.	1%	7%	23%	69%	92%
		ii. Give an overall interpretation of the main results, including heterogeneity across clusters in model performance, in the context of the objectives and previous studies (e.g., any comparisons to existing prediction models).	1%	7%	19%	73%	92%
	c. Implications	i. Discuss the potential use of the model and implications for future research, with a specific view to generalizability and applicability of the model across different settings or (sub)populations.	2%	8%	27%	62%	89%
		ii. Make clear how the AI/ML model and its outputs may be used and change clinical practice and work-up.	5%	12%	32%	51%	83%
7. Other	a. Supplementary information	i. Provide information about the availability of supplementary resources (e.g., study protocol, data sets).	2%	9%	25%	63%	88%
		ii. State whether and how the AI model and/or its code can be accessed, including any restrictions to access or re-use.	4%	6%	18%	71%	89%
	b. Funding	Give the source of funding and the role of the funders for the present study.	1%	1%	10%	87%	97%

From: Collins GS, Moons KGM, Dhiman P, et al. *BMJ* 2024;385:e078378. doi:10.1136/bmj-2023-078378
