# Supplementary material for: TRIPOD+AI statement: updated guidance for reporting clinical prediction models that use regression or machine learning methods
Source: BMJ. 2024 Apr 16;385:e078378. doi: 10.1136/bmj-2023-078378 (PMC11019967; doi:10.1136/bmj-2023-078378)
Supplement: Supplementary file 4 — Supplementary table 4: Aggregated TRIPOD+AI responses from Delphi round 2 [file colg078378.wt4.pdf]

Supplementary Table 4: Aggregated TRIPOD+AI responses from Delphi round 2

			Can be omitted	Possibly include	Desirable for inclusion	Essential for inclusion	Desirable + Essential
1. Title		Identify the study as developing or validating a multivariable prediction model, the target population, and the outcome to be predicted.	1%	4%	15%	80%	95%
2. Abstract		Provide a summary of objectives, study design, data sources, setting, participants, sample size, predictors, outcome, analytical methods, intended use of the prediction model, availability of the model, results, and conclusions.	0%	2%	14%	84%	98%
3. Introduction	a. Background	i. Explain the clinical context (including whether diagnostic or prognostic) and rationale for developing or validating the prediction model, including references to existing models.	1%	1%	15%	84%	99%
		ii. Explain the intended purpose (e.g., for prognosis or diagnostic predictions) and use for the prediction model in the context of the clinical pathway, including its intended users (e.g., healthcare professionals, patients, public).	1%	4%	25%	70%	95%
		iii. Describe the key population groups relevant to the disease outcome, including any known health inequalities between demographic groups.	4%	9%	42%	46%	88%
	b. Objectives	Specify the study objectives, including whether the study describes the development or validation of the model or both.	0%	3%	7%	90%	97%
4. Methods	a. Source of data	i. Describe the study design, source of data (e.g., randomized trial, cohort, routine care or registry data), separately for the development and validation datasets, and the rationale (and representativeness of the target clinical population) for using these data.	0%	1%	8%	91%	99%
		ii. Specify the key dates of the collected participant data, including start and end of participant accrual; and, if applicable, end of follow-up.	1%	5%	29%	65%	94%
	b. Participants	i. Specify key elements of the study setting (e.g., primary care, secondary care, general population) including the number and location of centres.	1%	6%	23%	70%	93%

Supplementary Table 4: Aggregated TRIPOD+AI responses from Delphi round 2

			Can be omitted	Possibly include	Desirable for inclusion	Essential for inclusion	Desirable + Essential
		ii. Describe the eligibility criteria for participants: how, where, and when potentially eligible participants were identified (e.g., symptoms, results from previous tests, inclusion in the registry, patient-care setting, location).	1%	3%	13%	83%	96%
		iii. Give details of any treatments received, and how they were handled during model development or validation, if relevant.	3%	12%	26%	59%	85%
	c. Data preparation	Describe any data pre-processing steps, including any cleaning, feature engineering, harmonisation, sampling, linkage, de-identification methods, augmentation and exclusions or changes made based on data quality data quality. Include details on whether data quality was similar across relevant demographic groups.	1%	4%	16%	80%	96%
	d. Outcome definition	i. Clearly define the outcome that is being predicted (and the time horizon), including how and when assessed, the rationale for choosing this outcome definition (if alternatives exist), and whether the method of outcome assessment is consistent across demographic groups.	0%	0%	5%	95%	100%
		ii. In case of outcome assessment requiring subjective interpretation, describe the qualifications of the outcome assessors and any measurement of inter- or intra- rater variability.	2%	9%	33%	56%	89%
		iii. Report any actions to blind assessment of the outcome to be predicted.	5%	9%	37%	49%	86%
	e. Predictors (features)	i. Clearly define all predictors, including how and when they were measured, any actions to blind assessment of predictors for the outcome and other predictors. Consider using supplementary material for large numbers of predictors.	1%	4%	14%	81%	95%
		ii. Describe the choice of initial predictors (e.g., literature, previous models, all available predictors) and any pre-selection of predictors prior to model building.	3%	6%	28%	63%	91%

Supplementary Table 4: Aggregated TRIPOD+AI responses from Delphi round 2

			Can be omitted	Possibly include	Desirable for inclusion	Essential for inclusion	Desirable + Essential
		iii. In case of predictor measurement requiring subjective interpretation, describe the qualifications of the predictor assessors and any measurement of inter- or intra-rater variability. Including any methods to mitigate or resolve discrepancies and materials needed for the predictor measurement. Consider whether the method of predictor measurement is consistent across demographic groups.	3%	9%	38%	50%	88%
	f. Class imbalance	If class imbalance was addressed, state why and how this was done, and any methods to recalibrate the model or the model predictions	2%	6%	23%	69%	92%
	g. Sample size	Explain how the study size was arrived at (separately for development and validation), including details of any sample size calculation. Provide a justification that the study size was sufficient to answer the research question.	1%	7%	25%	67%	92%
	h. Missing data	Describe in detail how missing data were handled (e.g., complete-case analysis, single imputation, multiple imputation, surrogate splits, pattern submodels). Provide reasons for omitting any data.	0%	2%	14%	84%	98%
	i. Analytical methods	i. Describe how the data were used in the analysis (e.g., any partitioning, and how this was done considering any sample size requirements, see item 4g). Provide details on how the data were used for any internal validation.	1%	4%	11%	85%	96%
		ii. Consider adding a diagram to illustrate the analytical processes.	8%	19%	49%	24%	73%
		iii. Depending on the type of model, describe how predictors were handled in the analyses (functional form, rescaling, transformation or any standardisation).	2%	3%	28%	67%	95%
		iv. Specify the type of model (and its rationale), how multinomial outcomes, survival (e.g., censored observations), or competing risks were handled (if appropriate), and all model-building procedures (including any data-driven predictor selection), and considering the following issues (where applicable).	1%	4%	17%	78%	95%

Supplementary Table 4: Aggregated TRIPOD+AI responses from Delphi round 2

			Can be omitted	Possibly include	Desirable for inclusion	Essential for inclusion	Desirable + Essential
		a) Describe the initialization of any model parameters (e.g., random, transfer learning).	1%	6%	31%	62%	93%
		b) Give a detailed description of the model, including inputs, outputs, and any intermediate layers and connections.	4%	11%	24%	61%	85%
		c) Describe the model training, including the procedure for any hyperparameter tuning, and number of models trained.	1%	4%	21%	74%	95%
		d) Describe the method of selecting the final model.	0%	2%	15%	83%	98%
		e) Describe any ensemble techniques (e.g., to combine model predictions).	2%	7%	28%	62%	90%
		f) Give details, including rationale, for any continual learning.	5%	12%	38%	45%	83%
		v. Describe how any heterogeneity in the model parameter values and model performance was handled and quantified across clusters (e.g., hospitals, countries). See TRIPOD-Cluster for additional considerations.	4%	5%	42%	49%	91%
		vi. Specify all measures used (and their rationale) to assess model performance (e.g., discrimination, calibration, net benefit) and, if relevant, to compare multiple models.	0%	5%	11%	84%	95%
		vii. Describe the methods for any analysis of prediction errors carried out. If no such analysis was carried out, explain why not.	3%	10%	31%	57%	88%
		viii. Describe any model updating (e.g., recalibration) arising from the validation, either overall or for particular populations (see item 4j on fairness) or settings.	2%	7%	30%	60%	90%
		ix. For external validation, describe how the model predictions were calculated (e.g., using code made publicly available).	3%	3%	21%	73%	94%
	j. Fairness	Provide details on how steps to recognise and avoid bias during the study, and whether decisions were taken during model development and validation to prioritise fairness and reduce health inequality.	5%	12%	28%	56%	84%

Supplementary Table 4: Aggregated TRIPOD+AI responses from Delphi round 2

			Can be omitted	Possibly include	Desirable for inclusion	Essential for inclusion	Desirable + Essential
	k. Model output and risk groups	Specify the output of the prediction model (e.g., probabilities, classification, risk grouping. Provide details (and rationale) for any risk groups/classifications, and how the thresholds were identified.	1%	2%	17%	81%	98%
	l. Development versus validation	For validation, identify any differences from the development (training) data in healthcare setting, eligibility, criteria, outcome, and predictors.	2%	2%	21%	74%	95%
	m. Ethical approval	Name the institutional research board or ethics committee that approved the study and provide a description of participant informed consent or an ethics committee waiver of informed consent.	3%	7%	16%	74%	90%
	n. PPI	Provide details of any patient and public involvement during the design, conduct, reporting (and interpretation) or dissemination of the study.	11%	20%	39%	30%	69%
5. Open Science	o. Protocol and registration	Provide details where the study protocol can be publicly accessed or state that a protocol was not prepared. Give details of registration and name of the registry.	6%	10%	33%	51%	84%
	p. Data	Provide details on availability of any data, or reasons for not sharing.	4%	10%	25%	61%	86%
	q. Code	Provide details of any software libraries, frameworks, or packages used in the work, as well as versions and licences under which they are available. Report the availability of any analytical code or previously unpublished code, provide the DOI or link if available. Give reasons for not sharing.	3%	11%	25%	62%	87%
6. Results	a. Participants	i. Describe the flow of participants through the study, including the number of participants with and without the outcome and, if applicable, a summary of the follow-up time. A diagram may be helpful.	3%	5%	15%	78%	93%
		ii. Report the characteristics overall and where applicable for each data source or setting, including the key dates, key predictors (including demographics), treatments received, sample size, number of outcome events, follow-up time, and amount of missing data. A table may be helpful. Report any differences across key demographic groups.	1%	4%	18%	77%	95%

Supplementary Table 4: Aggregated TRIPOD+AI responses from Delphi round 2

			Can be omitted	Possibly include	Desirable for inclusion	Essential for inclusion	Desirable + Essential
		iii. For validation, show a comparison with the development data of the distribution of important predictors/features (demographics, predictors, and outcome).	2%	8%	26%	63%	89%
	b. Model development	Specify the number of participants and outcome events in each analysis (e.g., model building, parameter tuning, validation).	2%	5%	14%	80%	94%
	c. Model specification	i. Provide details on the full prediction model (e.g., regression coefficients, input parameters, sharing of code/any dependencies), to allow predictions in new individuals, and to enable third-party evaluation and implementation.	2%	9%	18%	71%	89%
		ii. Give details on where the code (for the model) can be accessed, including any restrictions to access or re-use (e.g., freely available, proprietary). Report reasons for not sharing code.	4%	8%	24%	64%	88%
	d. Model performance	i. Report performance measures (with confidence intervals) for the prediction model.	0%	1%	5%	95%	100%
		ii. Report the results (e.g., model performance) in key subgroups (e.g., race, ethnicity, age, gender, sex) and any subgroups relevant to the outcome.	2%	9%	26%	63%	89%
		iii. Report results of any heterogeneity in model performance across clusters.	3%	8%	34%	55%	89%
		iv. Describe the results of any analysis of prediction errors and how errors were identified.	3%	8%	35%	54%	89%
	e. Model updating	Report the results from any model updating (including the updated model and subsequent performance), overall and for each cluster (e.g., hospital, if done).	2%	13%	28%	58%	86%
7. Discussion	a. Limitations	Discuss any limitations of the study (such as a non-representative sample, sample size, overfitting, missing data) and their effects on biases, statistical uncertainty, and generalizability.	0%	1%	8%	91%	99%

Supplementary Table 4: Aggregated TRIPOD+AI responses from Delphi round 2

			Can be omitted	Possibly include	Desirable for inclusion	Essential for inclusion	Desirable + Essential
	b. Interpretation	i. Give an overall interpretation of the main results, including issues of fairness, heterogeneity across clusters in model performance, in the context of the objectives and previous studies (e.g., any comparisons to existing prediction models).	1%	2%	17%	80%	97%
		ii. For external validation, discuss the results with reference to performance in the development data, and any other validation data.	1%	4%	20%	75%	95%
	c. Usability of the model in the context of current care	i. Explain how (and when in the clinical pathway) to use the prediction model including any onsite or offsite requirements (e.g., hardware/software requirements to permit third-party testing and implementation.	2%	12%	26%	60%	86%
		ii. Describe how poor quality or unavailable input data (e.g., predictors) should be assessed and handled when implementing the prediction model.	5%	15%	34%	46%	80%
		iii. Specify whether human-AI interaction will be required in the handling of the input data (e.g., steps required to go from the raw clinical data into a form usable by the model), and what level of expertise is required of users.	5%	14%	38%	44%	82%
		iv. Discuss the potential use of the model and implications and need for future research, with a specific view to generalizability and applicability of the model (e.g., across different settings or (sub)populations).	2%	8%	30%	61%	91%
8. Other	d. Non-technical summary	Consider providing a non-technical summary/box including the aim of the model (including the outcome being predicted), the population on whom it was trained and validated, and the results of model performance. Describe any future work required before the model can be implemented.	10%	17%	35%	38%	73%
	a. Conflicts of interest	State any conflicts of interest and financial disclosures for all authors.	2%	4%	5%	88%	93%
	b. Funding	Give the source of funding and the role of the funders for the present study.	2%	4%	14%	81%	95%

From: Collins GS, Moons KGM, Dhiman P, et al. *BMJ* 2024;385:e078378. doi:10.1136/bmj-2023-078378
